# Strophanthidin Attenuates MAPK, PI3K/AKT/mTOR, and Wnt/β-Catenin Signaling Pathways in Human Cancers

**DOI:** 10.3389/fonc.2019.01469

**Published:** 2020-01-17

**Authors:** Dhanasekhar Reddy, Preetam Ghosh, Ranjith Kumavath

**Affiliations:** ^1^Department of Genomic Science, School of Biological Sciences, Central University of Kerala, Kasaragod, India; ^2^Department of Computer Science, Virginia Commonwealth University, Richmond, VA, United States

**Keywords:** cardiac glycoside, strophanthidin, Na^+^/K^+^-ATPase, G2/M phase, apoptosis, autophagy

## Abstract

Lung cancer is the most prevalent in cancer-related deaths, while breast carcinoma is the second most dominant cancer in women, accounting for the most number of deaths worldwide. Cancers are heterogeneous diseases that consist of several subtypes based on the presence or absence of hormone receptors and human epidermal growth factor receptor 2. Several drugs have been developed targeting cancer biomarkers; nonetheless, their efficiency are not adequate due to the high reemergence rate of cancers and fundamental or acquired resistance toward such drugs, which leads to partial therapeutic possibilities. Recent studies on cardiac glycosides (CGs) positioned them as potent cytotoxic agents that target multiple pathways to initiate apoptosis and autophagic cell death in many cancers. In the present study, our aim is to identify the anticancer activity of a naturally available CG (strophanthidin) in human breast (MCF-7), lung (A549), and liver cancer (HepG2) cells. Our results demonstrate a dose-dependent cytotoxic effect of strophanthidin in MCF-7, A549, and HepG2 cells, which was further supported by DNA damage on drug treatment. Strophanthidin arrested the cell cycle at the G2/M phase; this effect was further validated by checking the inhibited expressions of checkpoint and cyclin-dependent kinases in strophanthidin-induced cells. Moreover, strophanthidin inhibited the expression of several key proteins such as MEK1, PI3K, AKT, mTOR, Gsk3α, and β-catenin from MAPK, PI3K/AKT/mTOR, and Wnt/β-catenin signaling. The current study adequately exhibits the role of strophanthidin in modulating the expression of various key proteins involved in cell cycle arrest, apoptosis, and autophagic cell death. Our *in silico* studies revealed that strophanthidin can interact with several key proteins from various pathways. Taken together, this study demonstrates the viability of strophanthidin as a promising anticancer agent, which may serve as a new anticancer drug.

## Introduction

Cancer denotes an assembly of diseases that share a collective overall phenotype, irrepressible cell progression, and proliferation which can affect any organ in humans (1). Cancer is the most prominent cause of death worldwide, with 1.7 million new cases per annum, and is likely to extend to 26 million by 2030 (2). Among all the cancers, breast cancer, and lung cancer are the most affected parts and are known to be malicious diseases in the world (3). Apart from this, liver cancer is also one of the most fatal cancers, having worldwide dominance, particularly in Asia and Africa. Even with the existing chemotherapy and other treatment modalities, cancer remains a challenging disease because of frequent deteriorations after therapy (4). Innumerable small molecules retaining antitumor activity have been discovered that includes cardiac steroids/cardiac glycosides (CGs), which are naturally derived organic compounds from plants' secondary metabolites that contain a sugar (glycoside) and an aglycone (steroid) moiety (5). CGs act by increasing the cardiac contractility by hindering the sodium—potassium—adenosine triphosphatase or sodium—potassium pump (Na^+^/K^+^-ATPase) of the plasma membrane (6). Apart from being involved in the membrane transporter function, Na^+^/K^+^-ATPase is also involved in cellular processes like cell survival and apoptosis along with contribution in cell signaling transduction pathways (7). Modern studies have publicized that CGs like digitalis, ouabain, digoxin, oleandrin, and bufalin have anticancer activity (8) and may serve as lead compounds for the improvement of various cancer treatments. Apoptosis with CGs is mainly associated with the inhibition of Na^+^/K^+^-ATPase and further generates downstream effector genes/proteins, which are related to cell growth and apoptosis by inhibiting the general protein synthesis, angiogenesis, anoikis sensitizers, and tumor growth (9). Apart from this, various reports suggest that several CGs such as ouabain and digoxin have a therapeutic effect on cancer by affecting topoisomerases I and II. Moreover, additional studies suggest that various types of tumor cells can show differential sensitivities to multiple CGs, and it is predicted that it may be because of differences in cellular contents of different cancer cells (10). However, the underlying antitumor contrivances of CGs have not been reported clearly. The activity of CGs depends on glycoside sugar regions, as well as the number of hydrogen/hydroxyl substituents and the linkage between oxygen and nitrogen in glycoside and aglycones. Structurally, strophanthidin is a monosaccharide CG with one aglycone portion and without any sugar unit (5). Strophanthidin is one of the less studied CGs, naturally derived from *Strophanthus kombe*. The action of strophanthidin was described to be similar to ouabain in heart failures. It has been reported that slight modification in either aglycone or sugar groups can significantly alter the efficiency of the CGs. A nitrogen link serves as an alternative for an oxygen link for sugar and steroid moiety, leading to the loss of activity (11). Some CGs (digitoxin) have been already approved as anticancer drugs through the drug repurposing approach. This motivates the need for further studies to explore the activity and underlying anticancer mechanism of strophanthidin due to its structural similarity to digitoxin and ouabain. In this study, the effect of strophanthidin was investigated on the signaling mechanism(s) such as MAPK, PI3K/AKT/mTOR, and Wnt/β-catenin pathways. Furthermore, we have investigated the role of strophanthidin in cell cycle arrest and in the expression of various proto-oncogenes, tumor suppressor genes (TSGs), and other transcription factors in three human cancer cell lines, that is, breast (MCF-7), non-small cell lung (A549), and hepatocellular (HepG2) carcinomas.

## Materials and Methods

### Chemicals and Reagents

Strophanthidin was procured from Sigma-Aldrich chemicals, while 3-(4,5-dimethylthiazol-2-yl)-2,5-diphenyltetrazolium bromide (MTT), Dulbecco's modified Eagle's medium (DMEM), fetal bovine serum (FBS), and propidium iodide were obtained from HiMedia Chemicals. + + + + + All the antibodies were purchased from Cell signaling technologies and Elabscience. Alexa Fluor 488 and ProLong Gold Antifade Mountant were procured from Thermo Fisher Scientific, 4′,6-diamidino-2-phenylindole (DAPI) was obtained from Roche Chemicals, and SYBR Green Master Mix was procured from Origin (India). Strophanthidin was stored as stock solution (10 mM) in DMSO in amber-colored glass containers at −20°C. Final working concentrations were prepared in the media before the experiment. The control contained the highest DMSO concentration (0.001%).

### Cell Culture

The human breast, lung, and hepatocellular carcinoma cell lines (MCF-7, A549, and HepG2) and normal lung and liver cells (L132 and WRL68) were obtained from the National Center for Cell Science (NCCS) (Pune, India). All these cells were cultured in DMEM supplemented with 2 mM l-glutamine, 10% FBS, 100 U/ml penicillin, and 100 mg/ml streptomycin. All cells were cultured in a 5% CO_2_ environment at 37°C. Peripheral blood mononuclear cells (PBMCs) were purchased from HiMedia (CL003-25). The cells were revived in RPMI medium supplemented with 10% FBS, 1% l-glutamine, and 1% penicillin–streptomycin solution. Approximately 1 × 10^4^ cells per milliliter were seeded in each well of a 96-well plate and incubated for 3–4 h. For checking the toxicity, 0.01–100 μM range of strophanthidin concentrations were used. Control cells were maintained in DMSO. All experiments were done with triplicates, and graphs were plotted in OriginPro 9.0.0.

### Cell Viability Assay

The antiproliferative effect of strophanthidin on MCF-7, A549, HepG2, and normal cells such as L132, WRL68, and PBMCs was determined by performing an MTT assay. About 4,000 cells per well were plated in a 96-well cell culture microplate and incubated overnight in complete media (DMEM containing 10% FBS with antibiotic) for cells to adhere to the plate. Cells were then treated with various concentrations ranging from 15 to 0.1 μM of strophanthidin for 24 h in serum-free media. An MTT assay was performed to identify cell viability. The absorbance of solubilized formazan was read at 570 nm and at the reference wavelength (non-specific readings) of 650 nm using a multimode plate reader (Epoch BioTek, USA).

### Comet Assay for DNA Damage

The alkaline comet assay for DNA damage was carried out according to Olive and Banáth (12), with slight modifications. The surface of the comet slides was scored with a diamond-tipped pen for better adhesion. Cell suspension of about 2 × 10^4^ cells per milliliter was mixed with low-melting agarose (Invitrogen, USA) and layered on dust-free comet slides without forming air bubbles. The air-dried slides were then placed in a prechilled neutral lysis buffer: 2% sarkosyl, 0.5 M Na_2_EDTA, and 0.5 mg/ml proteinase K (pH 10). The slides were then incubated at 37°C overnight. After overnight lysis, the slides were rinsed three times with an electrophoresis buffer for 20 min at room temperature, and electrophoresis was carried out for 25 min at 0.6 V/cm. The slides were then rinsed with distilled water and incubated with propidium iodide stain (2.5 μg/ml) for 20 min. This step was followed by destaining with distilled water, and the slides were visualized under the fluorescence microscope. Individual cells were visualized at 20X magnification in a Leica DMI3000 microscope with fluorescence attachments.

### Cell Cycle Analysis

Approximately 1 × 10^5^-10^6^ cells were seeded and incubated for overnight growth before treating with strophanthidin for 24 h. An appropriate number of cells were added to a conical tube and centrifuged at 1,000 rpm for 3 min. Then the cells were washed with chilled phosphate-buffered saline (PBS) and vortexed for 10 s to attain single-cell suspension. Then the cells were fixed with 4.5 ml of chilled ethanol (100%) for 30 min at 4°C. The cells were then rinsed with cold PBS to remove ethanol and then incubated with 10 μg/ml of RNase for 1–2 h in the dark at 37°C. After incubation, the fixed cells were stained with 0.25 μg/ml of propidium iodide for 30 min, and cell cycle distribution was measured by a flow cytometer (BD Biosciences).

### Gene Expression Studies Through Real-Time PCR

Total cellular RNA was isolated using the TRIzol^®^ reagent (Thermo Fisher Scientific, USA) as per manual instructions. The cells were treated with strophanthidin for 24 h, and the total RNA was isolated in the TRIzol reagent. Purity and concentrations were estimated using NanoDrop (Thermo Fisher Scientific, USA). A Verso complementary DNA (cDNA) synthesis kit was used for the synthesis of cDNA by following manufacturer instructions. A total of 2 μg of pretreated RNA was used for the synthesis of cDNA. Real-time quantitative PCR was performed using the Origin SYBR Green Master Mix (Origin, India) in the Roche LightCycler 480 system. RNA expression levels were normalized to that of GAPDH and calculated using the ΔΔCt method, and the log_2_ values were plotted in the graphs. All the primers used in this study are listed in Supplementary Table 1.

### Enzyme-Linked Immunosorbent Assay

Briefly, the cells were treated with lethal doses of strophanthidin for 24 h, and the total cell lysate was extracted with a RIPA lysis buffer (Thermo Fisher Scientific). Enzyme-linked immunosorbent assay (ELISA) antibodies for caspases 3, 7, 8, and 9 were purchased from Cell Signaling Technology. The experimentation was processed by following the manufacturer guidelines. Likewise, we performed ELISA to understand the role of strophanthidin in pathway activation, cellular growth, and apoptosis. We have used a PathScan^®^ MAP Kinase Multi-Target Sandwich ELISA Kit (Cell Signaling Technology, Cat# 7274), to understand the phosphorylation of Phospho-MEK1/2 (Ser217/Ser221), Phospho p38 MAPK (Thr180/Tyr182), Phospho p44/42 MAPK (Thr202/Tyr204), and Phospho-SAPK/JNK (Thr183/Tyr185). The absorbance was measured at 450 nm. The experiment was repeated thrice, and the obtained results were plotted in bar charts.

### Western Blot Analysis

Cells were initially treated with strophanthidin for 24 h. Treated cells were then harvested and lysed in lysis buffer containing 150 mM NaCl, 100 mM Tris (pH 8.0), 1% Triton X-100, 1% deoxycholic acid, 0.1% sodium dodecyl sulfate (SDS), 5 mM EDTA, 10 mM sodium formate, 1 mM sodium orthovanadate, 2 mM leupeptin, 2 mM aprotinin, 1 mM phenylmethylsulfonyl fluoride, 1 mM dithiothreitol, and 2 mM pepstatin A, along with a protease inhibitor cocktail (Roche, Lewes, Sussex, UK), on ice for 30 min. After centrifugation at 14,000 g for 15 min at 4°C, the supernatant was collected as total cellular protein content. The concentration of total proteins was estimated by the Bradford protein estimation assay. An equal concentration (30 μg) of total protein was resolved on 12–15% of SDS-polyacrylamide gel electrophoresis (PAGE) for different-sized proteins and transferred to a polyvinylidene difluoride (PVDF) membrane using the Trans-Blot Turbo Transfer System (Bio-Rad). The membrane was then blocked with 5% bovine serum albumin (BSA) and incubated with primary antibodies at 4°C overnight. After being washed three times with TBST containing 150 mM NaCl, 10 mM Tris, and 0.1% Tween 20 with pH 7.4, the membrane was incubated with a horseradish peroxidase (HRP)-conjugated secondary antibody for 1 h and washed with TBST for five to six times in 5-min intervals. Immunoreactive proteins were detected with a chemiluminescent ECL substrate (Bio-Rad) and quantified using the C-DiGit chemiluminescent western blot imaging system (LI-COR). Mean densitometry data from independent experiments were normalized to those of control experiments. Antibodies were procured as follows: caspases 3, 7, and 9 and PARP-1 were obtained from Cell Signaling Technology and Chk1, Chk2, cyclin D1, p53, AKT, p38, MEK1, mTOR, CDK6, SAPK/JNK, C-Myc, BAX, JAK, STAT3, GSK3α, β-catenin, Beclin 1, p62, LC3, PI3K, and GAPDH were obtained from Elabscience.

### Immunofluorescence

Immunofluorescence was done to understand the protein migration after treating with strophanthidin. Approximately 0.3 × 10^6^ cells were seeded on top of the coverslips in a 6-well plate. After 16 h, the cells were treated with strophanthidin and incubated for 24 h. After incubation, the cells were fixed with 4% paraformaldehyde for 20 min and again incubated with 0.1% Triton X for 20 min. After the coverslip was washed with 1X TBS four to five times, it was blocked with 5% BSA for 1 h. Then the coverslip was incubated with a primary antibody overnight at 4°C. The coverslip was then washed with 1X TBS and incubated in a secondary antibody (Alexa Fluor 488) for 2 h. Then the coverslip was washed and incubated with 0.1 μg/ml concentration of DAPI (Roche) for 20 min in the dark and washed five to six times with 1X TBS. Then the coverslip was transferred to clean glass slides coated with ProLong Gold Antifade Mountant (Invitrogen). Excess amount of Antifade was removed, and the slides were sealed with wax and observed under a fluorescent microscope with 40X magnification (Leica DMI3000, India).

### Molecular Docking With Discovery Studio

Using Discovery Studio V3.1 (built-in ligand preparation wizard), hydrogen atoms, probable tautomers, low-energy ring confirmers, and isomers were produced. Aromaticity was preserved for the compound, and pH (6.5–8.5) built ionization was applied. CHARMM was used for the energy minimization for dihedral angles and exact bond length. The 2D chemical structures of strophanthidin were downloaded from PubChem (6185), and it was exported to Discovery Studio V3.1 window for generating a 3D structure. Optimization was done using the CHARMM force field and root-mean-square (RMS) gradient energy (0.001 kcal/mol) with a default-parameter setup (13). Crystal structures of p38 alpha (PDB: 1OVE), NF-kβ (PDB: 1VKX), STAT3 (PDB: 1BG1), caspase 3 (PDB: 1NMS), Chk1 (PDB: 2E9P), Bcl-2 (PDB: 2O21), BCL-XL (PDB: 2W3L), MEK1 (PDB: 3VVH), and Chk2 (PDB: 2WTJ) protein structures were retrieved from the Protein Data Bank (PDB), and the retrieved structures were imported to the LibDock work environment. Later, the heteroatoms, co-factors, and unwanted water crystals were removed in the process of protein preparation. Along with this, protonation, ionization, energy minimization, and hydrogen bonds were added. The geometry was optimized with the CHARMM force field, and the modified protein was used for binding site definition from the “Edit binding site option from the receptor-ligand interaction toolbar.” Bound ligand binding position was used to create the active site and was found to have a 9.16-Å radius of active site. Docking was carried out by all prepared ligands, with each of the protein structures at the defined active site using LibDock from the receptor–ligand interactions toolbar. LibDock scores were used for all the docked ligand poses and further graded and grouped by names.

### Statistical Analysis

Statistical analysis was performed using OriginPro 9.0.0, and the significant variances between groups were determined by Student's *t*-test and/or ANOVA. Data were signified as mean ± standard error of the mean (SEM) from a minimum of three experiments. A *p* of <0.05 compared with the control was considered to be statistically significant.

## Results

### Effects of Strophanthidin on the Proliferation of Cancer Cells

**Figure 1:**
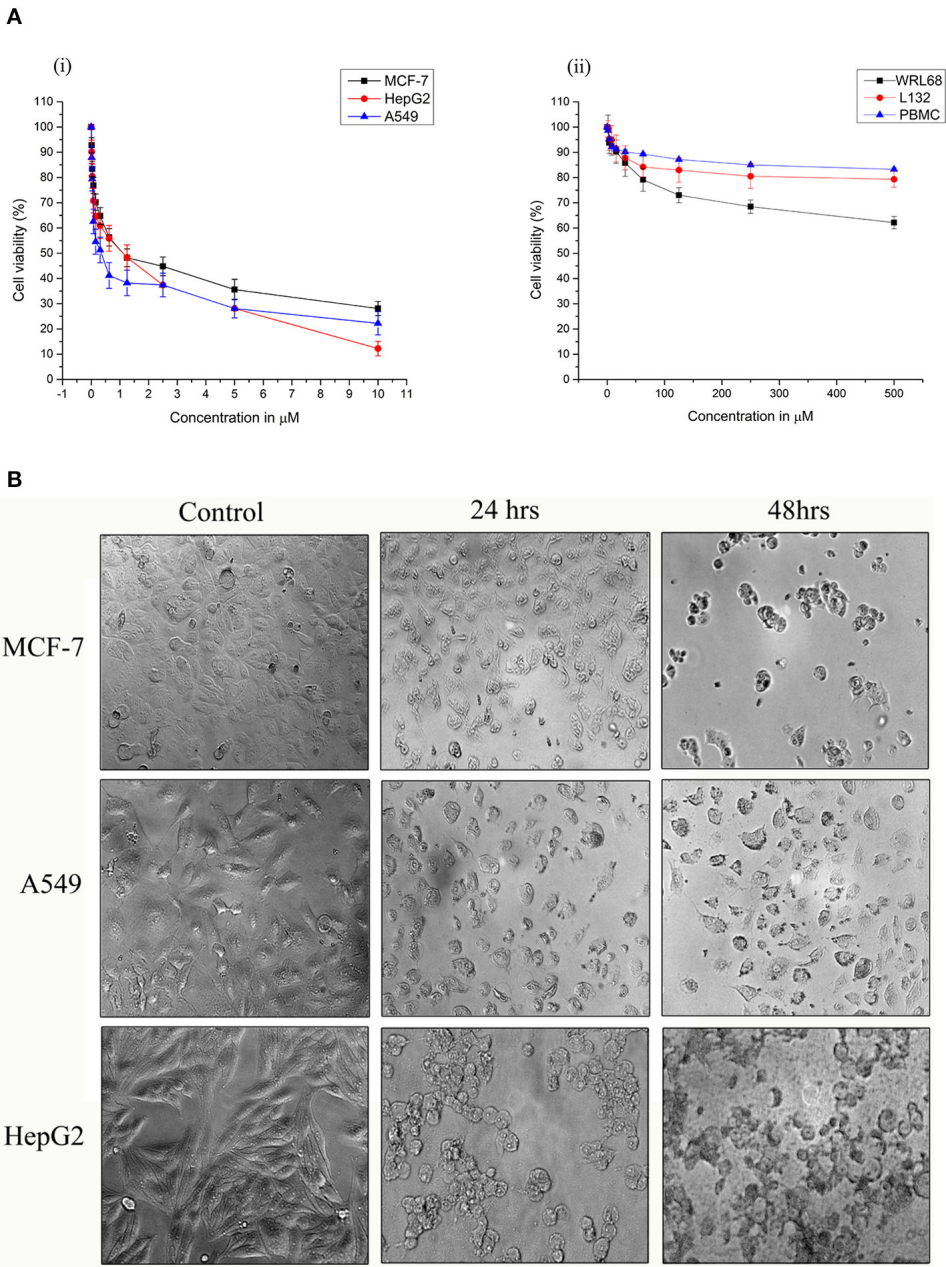
**(A)** Strophanthidin effectively suppresses the growth of human cancer cell lines. Cell viability of Strophanthidin in cancer cells (i) in comparison with normal cell lines (ii). Plots show mean values ± SE of quadruplicates with determinations of three or more experiments at *P* < 0.05. **(B)** MCF-7, A549, and HepG2 cells were treated with strophanthidin for 24 or 48 h. Morphological changes in the cells were observed. Representative images were obtained at 40X magnification. Scale bar: 50 μm.

Strophanthidin inhibited the proliferation in three different cancer cells, namely, MCF-7, A549, and HepG2, in a dose-dependent manner, and the obtained inhibitory concentrations (IC_50_) were shown in Figure 1A. It showed low values in A549 (0.529 ± 0.05 μM), high values in HepG2 (1.75 ± 0.02 μM), and moderate values in MCF-7 cells (1.12 ± 0.04 μM) [Figure 1A, (i)]. The nontoxic nature of this compound was evaluated in the non-malignant cells such as L132 and WRL68. However, we did not find any significant toxicity of strophanthidin in L132 and WRL68 at the IC_50_ concentrations of cancer cells (0.529–1.75 μM) and even up to Log2 difference of the IC_50_ concentrations [Figure 1A, (ii)]. We observed proliferation inhibition after treatment with strophanthidin for 24 h in all the cancer cells, under the microscope. The morphological observations have been checked in these concentrations at 24 and 48 h (Figure 1B). These data demonstrate that strophanthidin was effective at suppressing the growth of cancer cells and had no toxicity in normal cells. The structure of strophanthidin was compared with two known anticancer agents such as digitoxin and ouabain, and we found that the core structures of all these three compounds were the same (Supplementary Figure 1). All the chemical structures of compounds were drawn by using ChemDraw.

### Strophanthidin Does Not Show Significant Cytotoxicity in PBMCs

To evaluate the antiproliferative effect of strophanthidin in normal blood cells, we treated PBMCs with strophanthidin with a wide range from a high of 500 to 0.50 μM. At the concentrations of IC_50_ and at the difference of log_2_-fold, no inhibition or cell death were observed [Figure 1A, (ii)].

### Strophanthidin Treatment Causes Cell Death Through DNA Damage in Cancer Cells

Strophanthidin's contributions in inducing DNA damage were estimated through the comet assay. We observed the induction of DNA damage by the formation of comets after treatment with strophanthidin for 24 h in MCF-7, A549, and HepG2 cells (Supplementary Figure 2). This result suggests that strophanthidin mediates cell death by damaging DNA and that the movement of the tail increased rapidly in the case of treatment compared to control. The percentage of DNA is very high in the tail region compared to head regions, while the results were vice versa in the case of control. The percentage of tail movements and the percentages of DNA are shown in Table 1.

**Table 1:** Distance of comets traveled with and without treatment with strophanthidin for 24 h with IC_50_ concentrations.

**Cells**	**Total length of comet**	**Length of head**	**Length of tail**	**Head DNA (%)**	**Tail DNA (%)**	**Tail movement**	**Overall tail movement (OTM)**
MCF-7-control	34 ± 2.135	29 ± 3.479	5 ± 0.971	83.533 ± 3.457	16.46 ± 3.194	0.82 ± 0.251	0.55 ± 0.217
MCF-7-treated	96 ± 4.127	13 ± 1.473	83 ± 3.471	4.58 ± 0.247	95.41 ± 8.726	79.19 ± 3.457	40.21 ± 3.927
A549-control	28 ± 3.125	21 ± 3.847	7 ± 2.813	85.07 ± 6.438	14.92 ± 2.145	1.04 ± 0.439	1.80 ± 0.347
A549-treated	130 ± 6.945	61 ± 9.745	69 ± 5.761	58.23 ± 3.419	41.766 ± 3.821	28.817 ± 2.14	20.377 ± 3.127
HepG2-control	20 ± 3.179	13 ± 1.873	7 ± 1.574	76.27 ± 3.617	23.72 ± 2.841	1.66 ± 0.914	1.8 ± 0.617
HepG2-treated	72 ± 9.543	15 ± 1.817	57 ± 3.617	10.28 ± 0.517	89.71 ± 7.617	51.13 ± 3.146	24.98 ± 3.249

### Strophanthidin Induces Cell Cycle Arrests at the G2/M Phase in Cancer Cells

**Figure 2:**
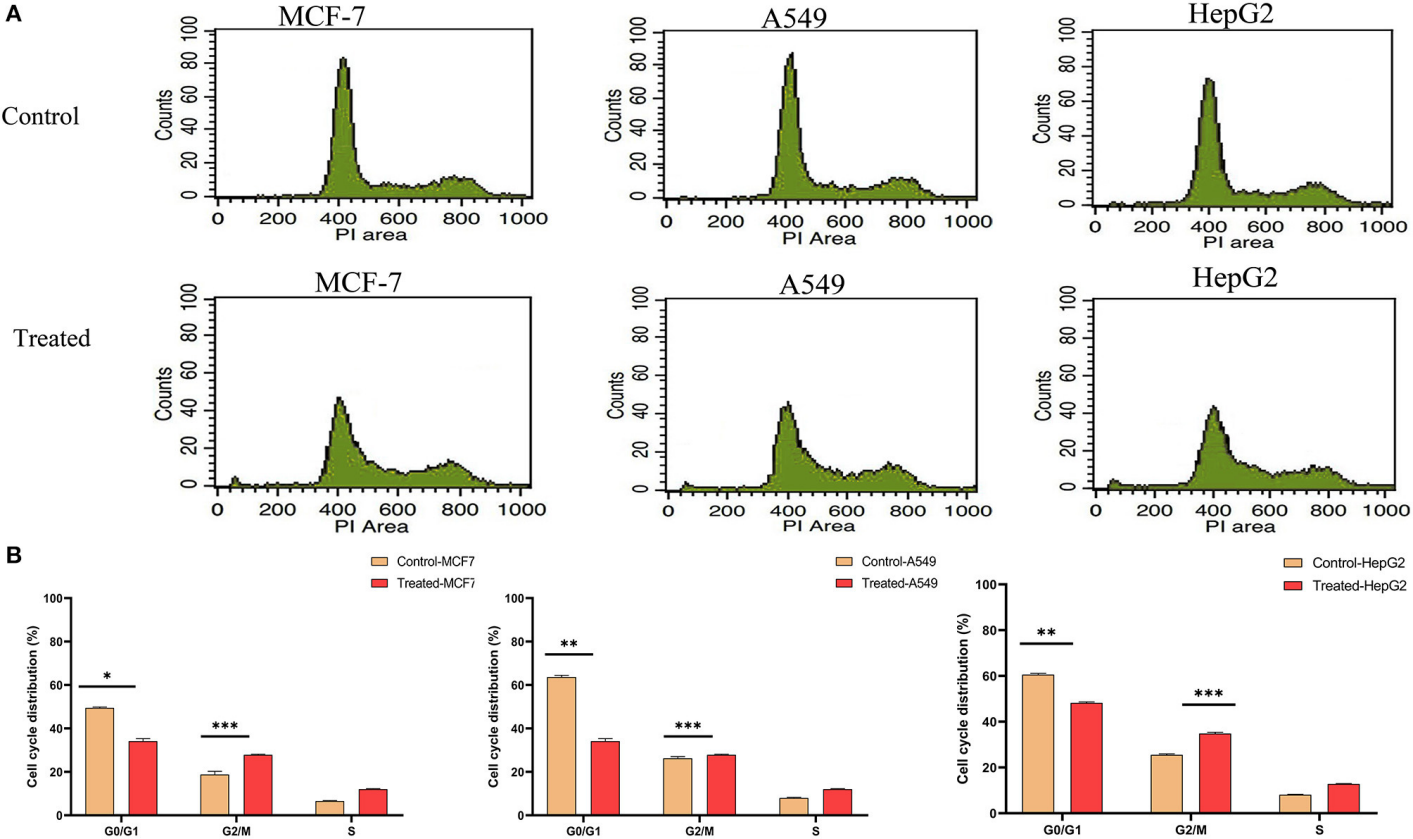
Strophanthidin induces cell cycle arrest at the G2/M phase. **(A)** Controls and treatments of MCF-7, A549, and HepG2 with strophanthidin and stained with propidium iodide, and the changes in cell cycle distribution were analyzed by flow cytometry (BD Bioscience). **(B)** Quantitative analysis and representation of flow cytometry data. Data are the mean values ± SE of at least three independent experiments performed in triplicates at *P* < 0.05. *p ≤ 0.05, **p ≤ 0.01, ***p ≤ 0.001.

As strophanthidin showed clear cytotoxicity and DNA damage in three cancer cell lines used in the current study, we evaluated whether these effects correlated with the events of the cell cycle. All the cells were exposed to serum starvation for 72 h and then treated with strophanthidin with lethal concentrations of IC_50_ for MCF-7 (2 μM), A549 (1 μM), and HepG2 (2.5 μM), and all the experiments were carried out with these concentrations. Treated cells were incubated for 24 h with serum. While releasing the cells, we observed that more number of cells were arrested in G2/M (Figures 2A,B). In MCF-7, the control gated 62.74% cells at G0/G1, but the treatment was restricted to 44.28%. Likewise, in A549 cells, a total of 63.38% of cells were gated at G0/G1, whereas the treatment was limited to 44.22%. In HepG2 cells, 61.33% cells were gated in G0/G1, whereas the treatment was regulated to 43.66%. In the case of the G2/M phase, the total number of cells gated for MCF-7 control was 18.82%, whereas the treatment raised this to 37.84%. In the case of A549, the control gated cells were 26.25%, and in treatment, they increased to 27.85%. In HepG2 cells, the total number of cells gated in control for the G2/M phase was 25.51%, and we have observed 34.82% of cells in the treatment conditions. This indicated that strophanthidin can arrest cell cycle at the G2/M phase in all studied cancer cells. Along with the studies on cell cycle, we analyzed the percentage of dead cells in treatments compared to control cells. In MCF-7 cells, the percentage of dead cells in control was 2.27%, but in the case of treatment, the percentage of dead cells was doubled and reached 4.87%. A549 control cells contain only 2.23% of dead cells, but with strophanthidin treatment, a total of 4.22% of dead cells were observed in the total gated cells. In the case of HepG2 cells, dead cell percentage in control was 3.77%, but in the case of treatment, the percentage increased to 5.66%. In order to validate these results, we further investigated important genes and proteins that can mediate cell cycle progression, which includes cyclin D1, Chk1, Chk2, and CDK6.

### Strophanthidin Inhibits Expression of G2/M Cell Cycle Regulator, MAPK, and PI3K/AKT/mTOR Pathway Genes

**Figure 3:**
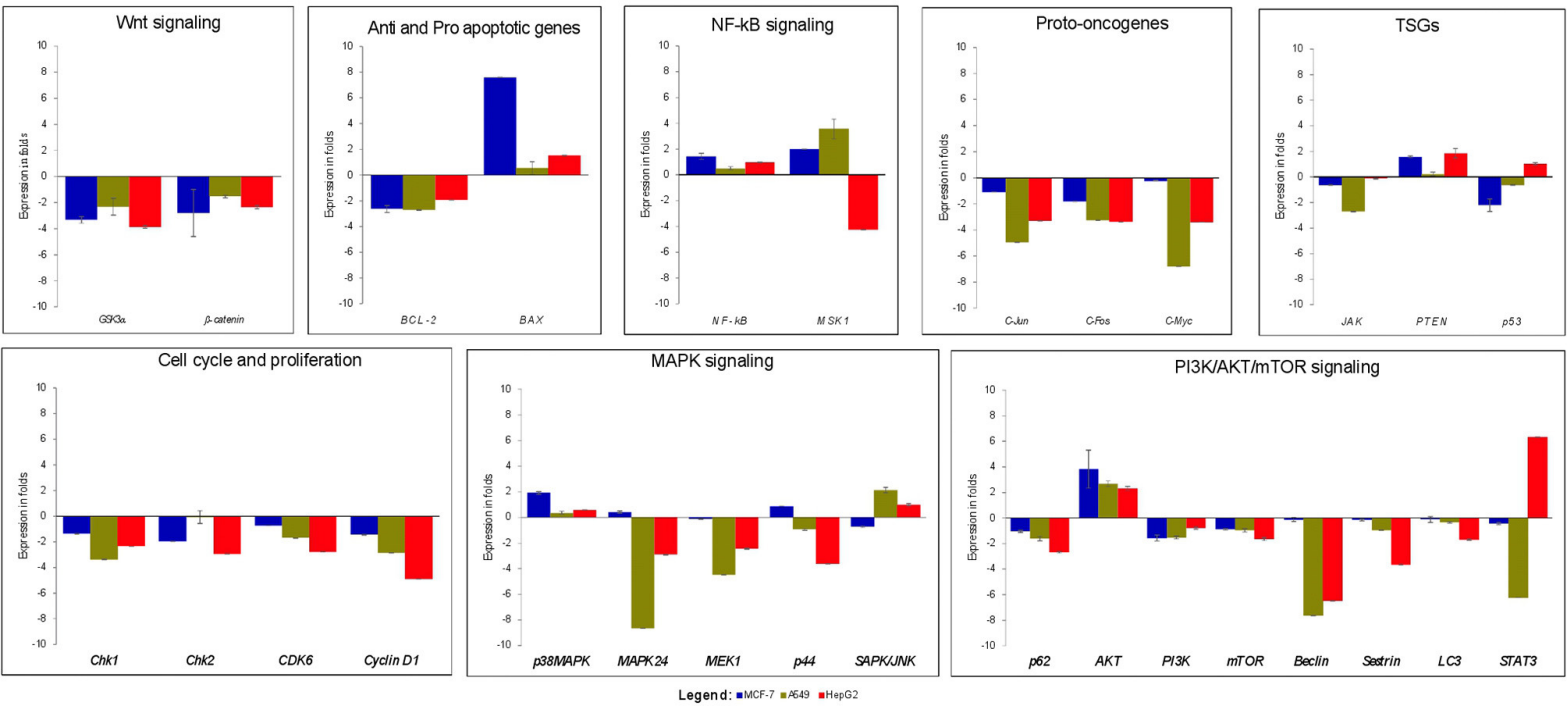
Gene expression analysis of various genes related to cell death and survival from various signaling transduction pathways; GAPDH was used as internal control. MCF-7 cells were treated with 2 μM concentration of Strophanthidin for 24 h, and the expressions were normalized with GAPDH. Gene expression analysis of A549 cells induced with 1 μM concentration of strophanthidin for 24 h, and the obtained results were normalized with GAPDH. Real-time PCR gene expression analysis of HepG2 cells treated with 2.5 μM concentrations of strophanthidin, and the expressions were normalized to GAPDH. All the expressions were analyzed with the 2–ΔΔCt method, and the obtained results are statistically significant (*n* = 3 and *P* ≤ 0.01).

Real time-PCR was performed to study the effects of strophanthidin in altering the expression level of various genes involved in cell signaling. We initially checked the expression of proto-oncogenes (*c-Myc, c-Jun*, and *c-Fos*) in strophanthidin treatment and observed significant downregulation. Further, we extended our study to identify the regulation of cell cycle-dependent genes such as *cyclin D1, Chk1, Chk2*, and *CDK6* and observed inhibition in their expressions. We analyzed the role of strophanthidin treatment in several biochemical signal transduction pathways such as the NF-κB signaling pathway and observed cell-specific expression. In MCF-7 and A549, genes were upregulated, whereas, in HepG2, overexpression of NF-κB inhibited the expression of *MSK1*. In addition to this, the role of strophanthidin in PI3K/AKT/mTOR autophagy was also checked. To asses this effect, we analyzed the expressions of *p62, AKT, PI3K, mTOR, Beclin, Sestrin*, and *LC3* and observed the significant downregulation in *p62, LC3, Beclin, Sestrin, mTOR*, and *PI3K* in all cancer cells and upregulation of *AKT*. Further, for studying the effect of strophanthidin in MAPK signaling, *p38MAPK, MEK1, MAPK24, SAPK/JNK*, and *p44* genes were selected. Initiator gene *p38* is highly expressed, and *MEK1* is deregulated in all the cells used in this study. The expressions of *MAPK24* and *p44* were also in a cell-specific manner; that is, only breast cells showed upregulation of these genes, whereas lung and liver cells showed downregulation. *SAPK/JNK* also showed the differential expression by undergoing downregulation in MCF-7 and upregulation in A549 and HepG2 cells. Subsequently, we checked the effect of strophanthidin in the expression of the JAK-STAT pathway by analyzing the expression levels of *JAK* and *STAT3* genes. We found downregulations of both genes irrespective of the type of cancer cells. For understanding the effect on Wnt/β-catenin pathway, we checked the expression levels of *Gsk3*α and β*-catenin*. Substantial dysregulation of *Gsk3*α was observed, which further suppressed the expression of β*-catenin*. In addition to this, we analyzed the expression levels of TSGs (*PTEN* and *p53*), a pro-apoptosis gene (*BAX*), and an antiapoptotic gene (*Bcl-2*). We observed cell-specific expressions, as *PTEN* is dysregulated in MCF-7 and HepG2 and overexpressed in A549. In the case of *p53*, we observed downregulation in MCF-7 and A549 as opposed to upregulation in HepG2 cells. At the same time, consistent overexpression and dysregulation were observed for *BAX* and *Bcl-2*, respectively, in all the cancer cells (Figure 3).

### Strophanthidin Dysregulates the Expression of Checkpoint and Cyclin-Dependent Kinases in Cancer Cells

**Figure 4:**
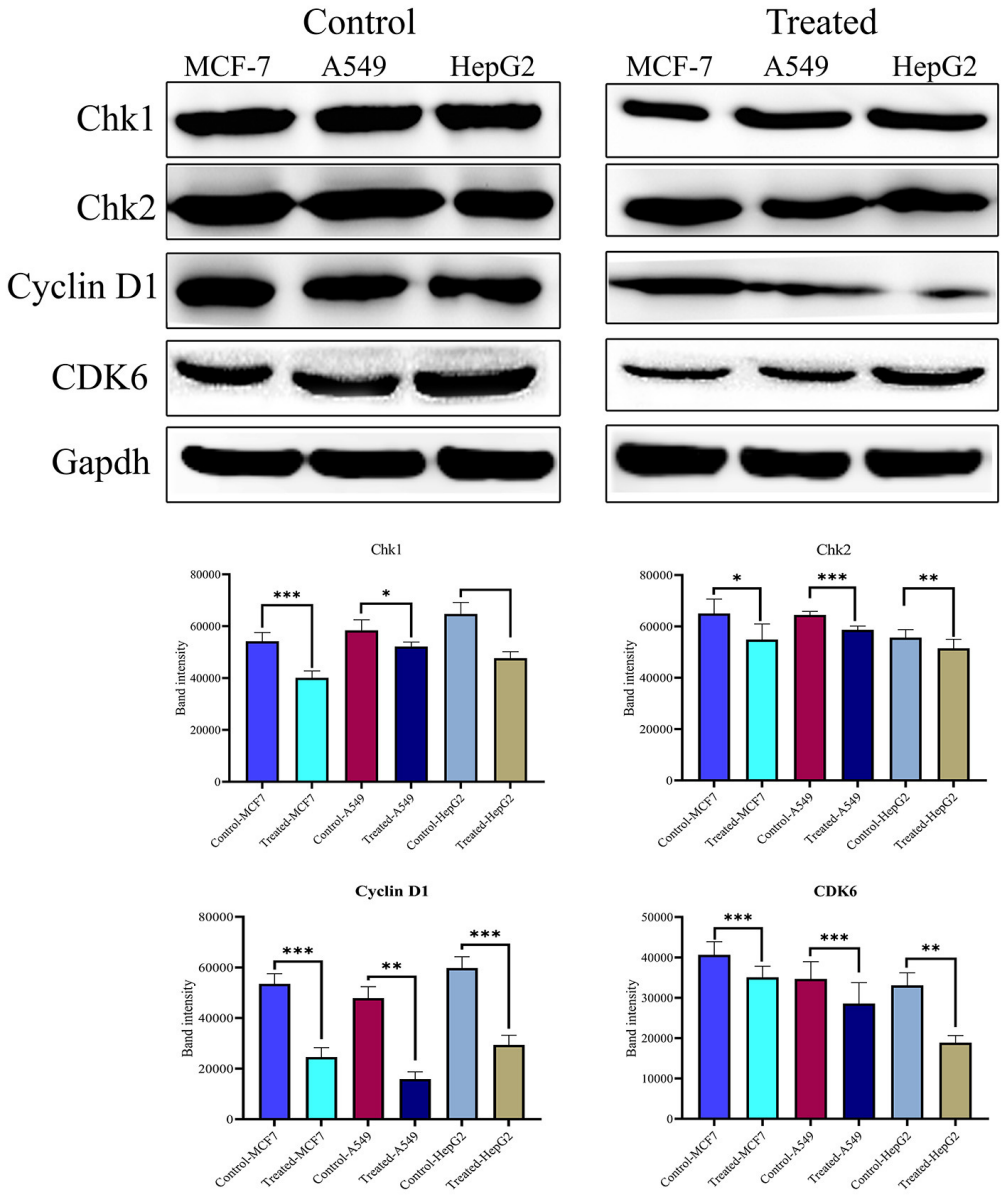
Western blot expressions of target proteins with strophanthidin treatment. Expression of cell cycle-regulating proteins such as Chk1, Chk2, CDK6, and cyclin D1 in three cancer cell lines and statistical analysis of cell cycle-regulating proteins in strophanthidin-treated cancer cells. Blots were compared with those of GAPDH expression to compare equal loading of samples. Representative blots from three independent experiments are shown (*P* < 0.05). *p ≤ 0.05, **p ≤ 0.01, ***p ≤ 0.001.

The cell cycle plays a critical role in cancer cell survival and death. To determine whether strophanthidin at tested concentrations induces the expressions of several checkpoints and cyclin-dependent kinase proteins, we performed and analyzed western blot experiments for cyclin D1, Chk1, Chk2, and CDK6. We found that strophanthidin inhibits the expression of checkpoint and cyclin-dependent kinases in three cancer cells compared to untreated controls (Figure 4).

### Strophanthidin Shows Caspase-Dependent Cell Death in Cancer Cell Lines

**Figure 5:**
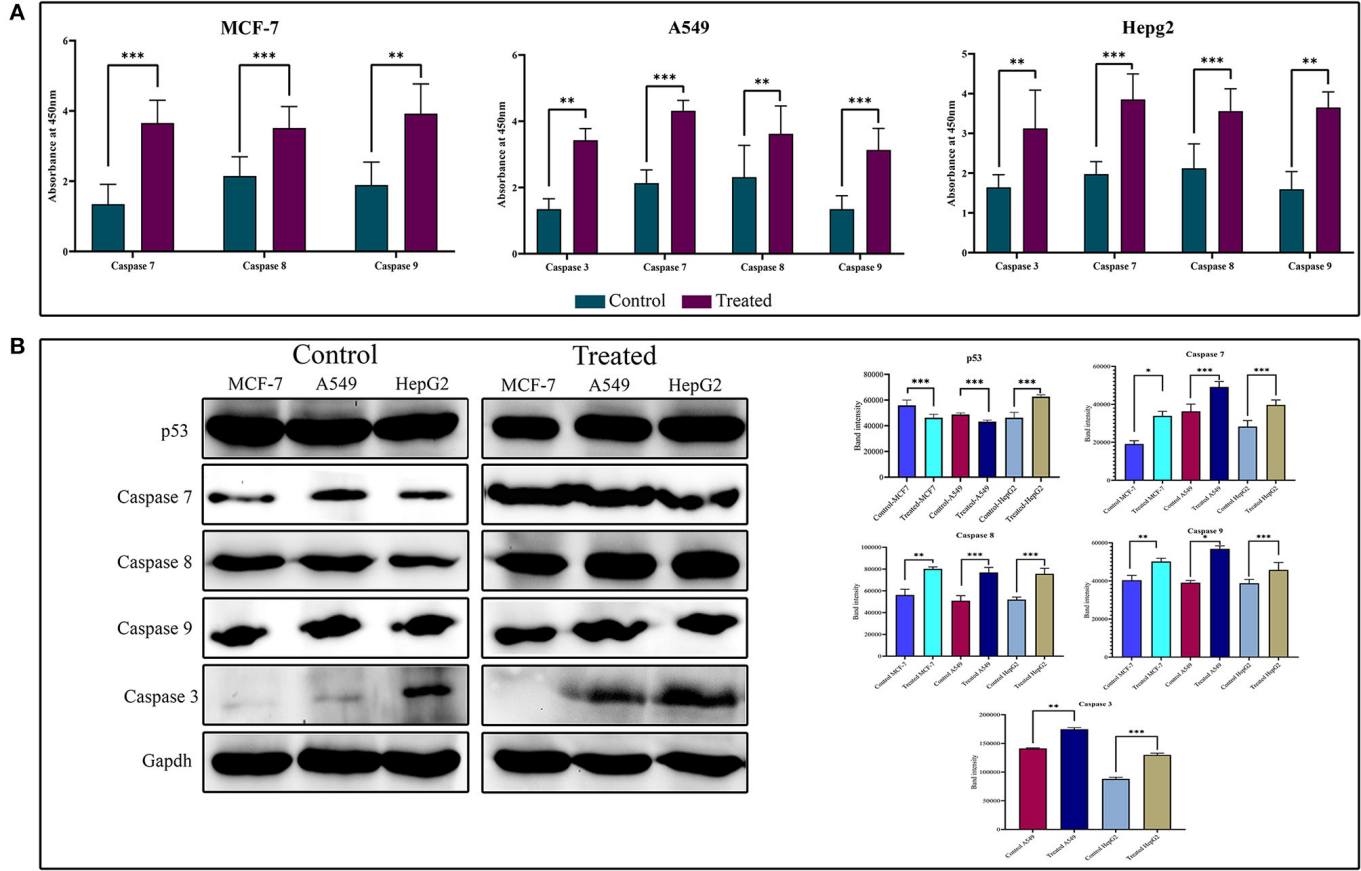
**(A)** Enzyme-linked immunosorbent assay (ELISA) for the expression analysis of caspases on strophanthidin treatment. Overexpressions of caspases 3, 7, 8, and 9 in strophanthidin-treated cells along with controls were observed in strophanthidin-induced MCF-7, A549, and HepG2 cells. **(B)** MCF-7, A549, and HepG2 cells were treated with lethal doses of Lanatoside C for the indicated times, and lysates were prepared. Western blot analysis was performed with antibodies to detect activation caspases 3, 7, 8, and 9. Also, the differential expression of p53 was observed. Experiments were performed in triplicates (*n* = 3), and the *P* is <0.05. *p ≤ 0.05, **p ≤ 0.01, ***p ≤ 0.001.

To show the activity of strophanthidin as an anticancer agent, we determined the expression of proteins involved in apoptosis such as BAX, proto-oncogenes like c-Myc, caspases, and PARP in tumor cells (Figure 5A). Our results suggest that strophanthidin treatment in cancer cells led to the overexpression of the initiator caspase 9 and further led to the activation of execution caspases including caspases 3 and 7, which eventually induced apoptosis. Along with that, we investigated the expression of caspase 8 in the treatment conditions compared to controls and identified that this protein is highly upregulated in the treatments compared to control cells. On the other hand, we have observed the expressions of c-Myc (proto-oncogene) downregulation and BAX (pro-apoptotic protein) upregulation in MCF-7, A549, and HepG2 cells. A type of cell death initiated by the JAK-STAT pathway was also observed by checking the expressions of JAK, STAT3, and p53 proteins. Strophanthidin at its inhibitory concentrations showed cell-specific expression changes in HepG2 cell lines. Both transcription factor STAT3 and tumor suppressor p53 showed significant upregulation, whereas they showed downregulation in MCF-7 and A549 cells. JAK protein showed consistent downregulation in the three cell lines (Figure 5B and Supplementary Figure 3). All the obtained expressions were normalized to GAPDH, and the graphs were plotted with mean values.

### Strophanthidin Exerts Changes in the Expression of PI3K/AKT/mTOR Signaling

**Figure 6:**
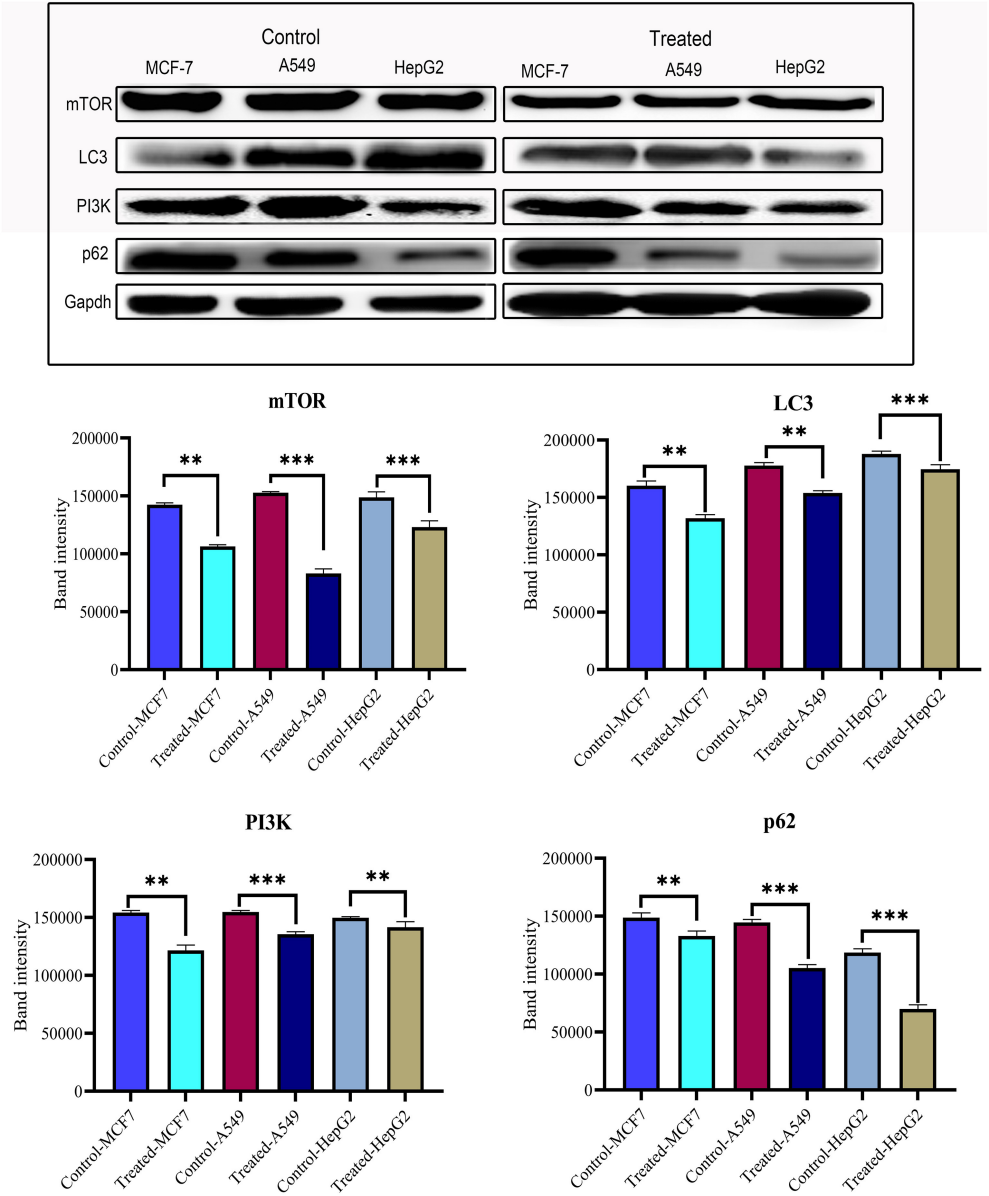
Western blot and statistical analysis of proteins from PI3K/AKT/mTOR signaling. Overexpression of AKT and downregulation of mTOR, LC3, PI3K, p62, and Beclin 1 have also been observed. Blots were compared with those of GAPDH expression to compare equal loading of samples. Representative blots from three independent experiments are shown (*P* < 0.05). **p ≤ 0.01, ***p ≤ 0.001.

After confirming the anticancer potential of strophanthidin, we recognized some of the crucial pathways to associate such kind of effects. MCF-7, A549, and HepG2 cells were incubated with strophanthidin with tested concentrations for 24 h, and we evaluated the protein expressions related to PI3K/AKT/mTOR pathways that can impact apoptosis, cell cycle, cell survival, and autophagy. In this study, we selected the crucial proteins such as AKT, mTOR, LC3, Beclin 1, PI3K, and p62. After analyzing the results, we identified the overexpression of AKT in all the cell lines related to the untreated cases. A consistent downregulation was observed in the case of mTOR, LC3, Beclin 1, PI3K, and p62, supporting the gene expression studies. These expressions specify the role of strophanthidin in cell death and autophagy signaling. The obtained expressions were normalized to GAPDH expression, and mean densitometry graphs were plotted with standard errors (Figure 6 and Supplementary Figure 3).

### Strophanthidin Alters the Expressions in the MAPK Signaling and Wnt/β-Catenin Pathway

**Figure 7:**
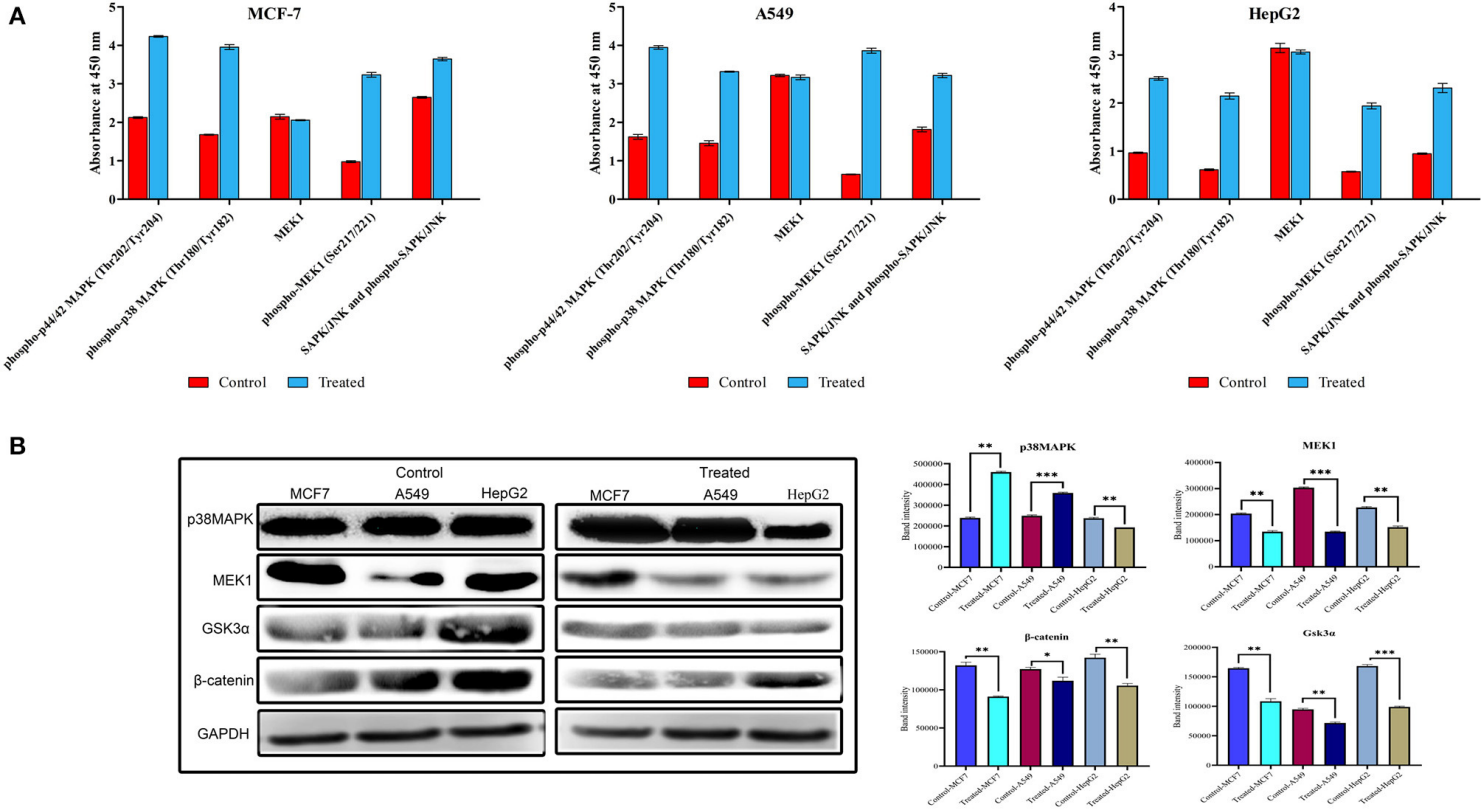
**(A)** Detection of relative Phospho-MEK, p38 MAPK, p44/42, and SAPK/JNK levels in MCF-7, A549, and HepG2 cells following stimulation with strophanthidin. All the cancer cells were treated with strophanthidin for 24 h. Cell lysates were analyzed using rabbit Phospho-MEK (S217/S221), Phospho p38 MAPK (Thr180/Tyr182), Phospho p44/42 MAPK (Thr202/Tyr204), and Phospho-SAPK/JNK (Thr183/Tyr185) and total MEK1 with enzyme-linked immunosorbent assay (ELISA) (category # 7274). **(B)** Western blot and statistical analysis of proteins from MAPK and Wnt/β-catenin signaling. Cell-specific expression of p38MAPK and consistent downregulation of MEK1 were observed. Both Gsk3α and β-catenin are downregulated in strophanthidin-induced cancer cells. Blots were compared with those of GAPDH expression to compare equal loading of samples. Representative blots from three independent experiments are shown (*P* < 0.05). *p ≤ 0.05, **p ≤ 0.01, ***p ≤ 0.001.

To investigate the role of strophanthidin in the phosphorylation event of MAPK signaling, we performed ELISA with the phosphorylated antibodies of MEK1, p38MAPK, p44/42, and SAPK/JNK. Cancer cells were treated with strophanthidin for 24 h, and the total cell lysates were analyzed using Phospho-MEK1/2 (Ser217/Ser221), Phospho p38 MAPK (Thr180/Tyr182), Phospho p44/42 MAPK (Thr202/Tyr204), and Phospho-SAPK/JNK (Thr183/Tyr185). After strophanthidin treatment, a robust and rapid phosphorylation of MEK1, p38MAPK, p44/42, and SAPK/JNK was detected in parallel with total MEK1 and p38MAPK levels (Figure 7A). To determine the role of strophanthidin on MAPK signaling, we have squared the expressions of p38 MAPK, MEK1, and SAPK/JNK. In the case of p38MAPK, differential cell-dependent expressions were observed. In the case of HepG2 cell, this protein was inhibited, whereas it exhibited consistent upregulation in the two other cell lines. The reason for this differential expression may be due to the activity of CGs in general protein synthesis inhibition. We also observed significant downregulation in the expressions of MEK1, while in SAPK/JNK, we found strong upregulation (Figure 7B). The Wnt/β-catenin pathway displays a significant role between cell survival and apoptosis. In the current study, we have checked the expression regulations of Gsk3α and β-catenin from the Wnt/β-catenin signaling pathway. Inhibitory concentrations of strophanthidin displayed consistent dysregulation of both of these proteins, supporting the gene expression studies (Figure 7B). All the protein expressions were normalized to internal control, and mean densitometry graphs were plotted.

### Immunofluorescence Analysis-Based Confirmation of Pathways Attenuated by Strophanthidin

**Figure 8:**
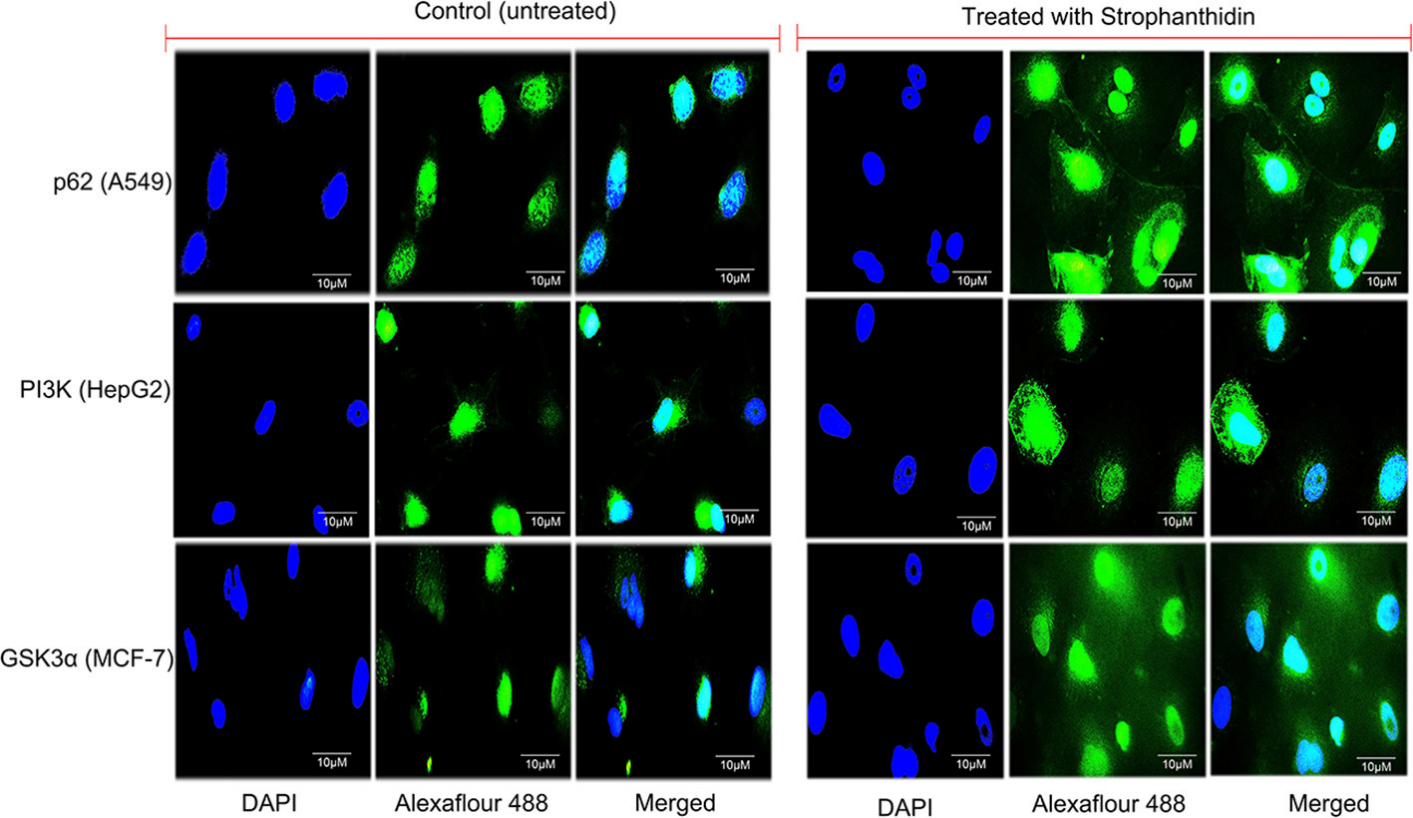
Immunofluorescence imaging for the analysis of protein localizations in strophanthidin-induced A549 cells (p62), MCF-7 cells (GSK3α), and HepG2 cells (PI3K) was observed in comparison with control. Scale bar: 10 μM.

We performed immunofluorescence to identify the underlying mechanism of the antiproliferative activity of strophanthidin in MCF-7 (Supplementary Figures 4A–G), A549 (Supplementary Figures 5A–G), and HepG2 (Supplementary Figures 6A–G). Subcellular localization describes the function and presence of proteins inside the cells; therefore, we initiated this investigation and analyzed the localization of several proteins from cell cycle regulation such as Chk1, Chk2, CDK6, and cyclin D1; we found that strophanthidin treatment can change protein localization in the cytoplasm and within the membrane as well as nucleus, whereas it was only present in the nucleus in untreated cells. Also, we checked the localizations of two crucial proteins, that is, pro-apoptotic protein (BAX) and proto-oncogene encoding protein (c-Myc), which play a foremost role in apoptosis. Strophanthidin treatment showed overexpression of BAX and low expression in c-Myc. We then checked the role of some important proteins from different biochemical signal transduction pathways: Gsk3α and β-catenin from Wnt/β-catenin signaling; JAK, STAT3, and p53 from JAK-STAT signaling; p38MAPK, MEK1, and SAPK/JNK from MAPK signaling; and AKT, mTOR, LC3, p62, and PI3K from PI3K/AKT/mTOR signaling in three cancer cells. Taken together, the obtained results suggested that strophanthidin could modulate the protein localization from the nucleus to the membrane as well as to the cytoplasm. Here, we have shown the localization of p62 from A549 (control and treated), PI3K from HepG2 cells (control and treated), and GSK3α from MCF-7 cells (control and treated) (Figure 8).

### Molecular Docking Analysis Shows Strophanthidin Can Potentially Inhibit Multiple Cancer Targets

**Figure 9:**
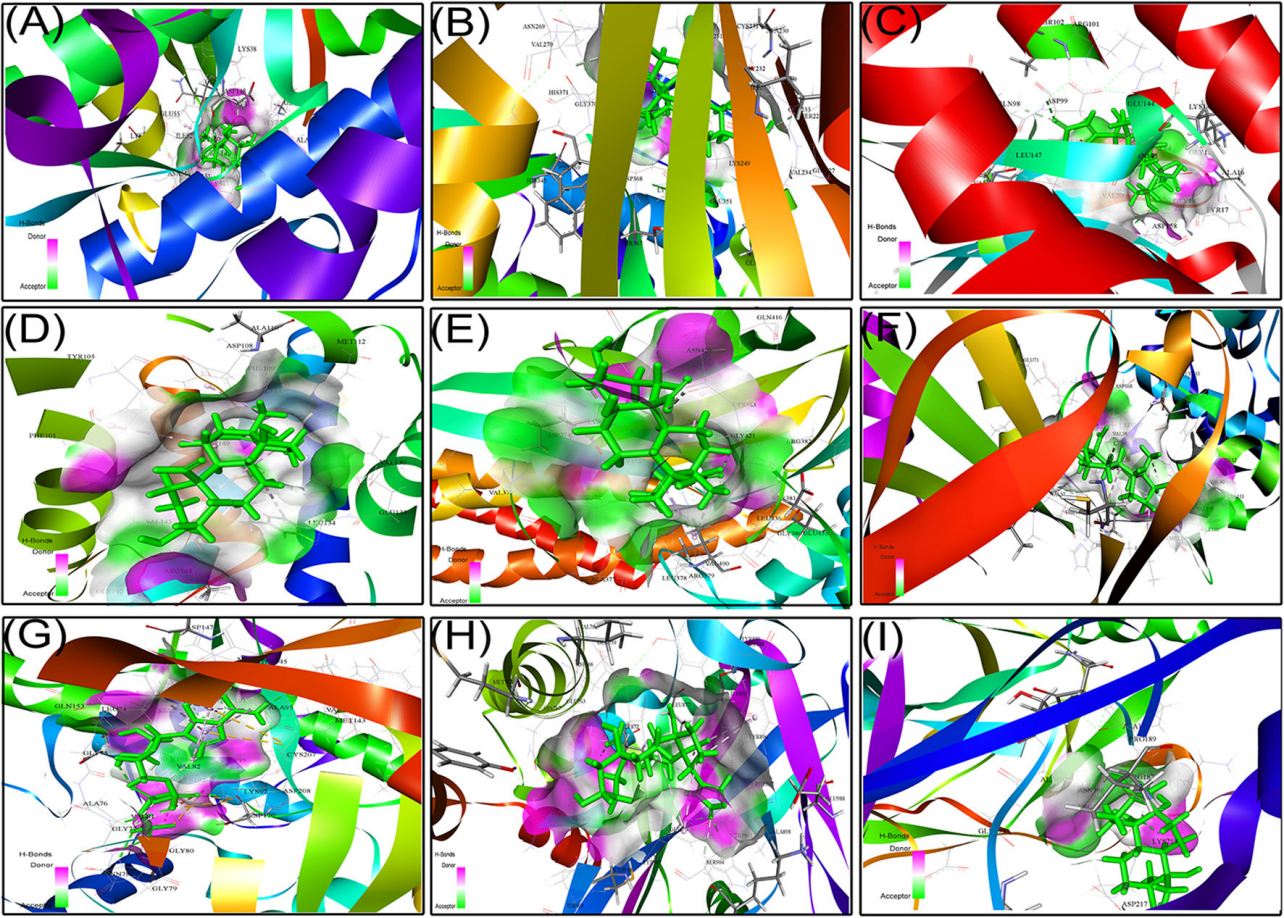
Protein–ligand docking complex of **(A)** Chk1, **(B)** Chk2, **(C)** cyclin D1, **(D)** Bcl-2, **(E)** STAT3, **(F)** p38MAPK, **(G)** MEK1, **(H)** PARP, and **(I)** NF-kβ with strophanthidin.

We next studied the interactions of strophanthidin with various cell cycle-regulating proteins like checkpoints and cyclin-dependent kinases (Chk1, Chk2, and cyclin D1). It was observed that MET42 interacts with strophanthidin and forms a hydrogen bond with Chk1 with a LibDock score of 84.1472. Chk2 forms four hydrogen bonds with strophanthidin at GLU233, VAL270, ASP347, and HIS371, with a LibDock score of 90.4444. Then, we checked the interactions with cyclin D1 and found that GLN98 and ASP99 interacts with the ligand to form hydrogen bonds with a LibDock score of 87.7488. We further extended our study to identify the binding mechanisms of strophanthidin with antiapoptotic proteins such as Bcl-2 and Bcl-xl. Strophanthidin packs against the residues PHE150, ASP61, ARG65, PHE71, and SER75, to form hydrogen bonds for Bcl-2 and Bcl-xl with scores of 98.77 and 78, respectively. The docking phase of STAT3 showed that strophanthidin can bind to the DNA binding domain and pack against the residues ARG379 and HIS437 and form hydrogen bonds with the highest LibDock score of 106.562. The molecular docking level of MAPK signal transduction pathway proteins such as p38 alpha and MEK1 showed good binding with strophanthidin and packing against ALA111, GLY77, ASN78, LYS97, GLU144, MET146, and SER194, by forming hydrogen bonds with LibDock scores of 110.982 and 66.5941, respectively. Finally, we checked the interactions of strophanthidin with apoptosis-regulating proteins caspase 3 and PARP. We found fitting molecular docking with both target proteins. Residues ASN208, TRP214, HIS862, GLY863, ASN868, and SER904 interacted with strophanthidin to form hydrogen bonds with LibDock scores of 58.1 and 137.42 for caspase 3 and PARP, respectively. The binding pockets of the ligand–protein complex are available in Figure 9. Other than hydrogen bond-forming residues, many other residues were also noted to be interacting, which are shown in Supplementary Figure 7, and the residues are reported in Table 2.

**Table 2:** Ligand interactions of strophanthidin with various cell signaling proteins from different pathways and the residues which are forming hydrogen bonds along with amino acids at 4-Å distance.

**PDB ID**	**Protein name**	**LibDock score**	**No. of hydrogen bonds**	**Interacting residues**
1BG1	STAT3	106.562	4	**H bonds:** ARG379, HIS437
				**Interacting residues:** ASP369, SER372, GLY373, ASP374, VAL375, ALA377, LEU378, GLY380, SER381, ARG382, LYS383, GLN416, ASN420, GLY421, LEU436
CDK4 or CYCLIN D1	Cdk4	87.7488	2	**H bonds**: GLN98, ASP99
				**Interacting residues:** ILE12, GLY13, VAL14, GLY15, TYR17, GLY18, THR19, VAL20, LYS35, ASP97, ARG101, GLU144, ASN145, LEU147, ASN158
1WOK	PARP	137.42	4	**H bonds:** HIS862, GLY863, ASN868, SER904
				**Interacting residues:** GLN759, GLU763, ASP766, ASN767, TRP861, SER864, ARG865, TYR889, ILE895, TYR896, TYR897, ALA898, TYR907, HIS909
2WTJ	CHK2	90.4444	3	**H bonds:** GLU233, VAL270, ASP347, HIS371
				**Interacting residues:** GLY227, SER228, GLY229, CYS231, GLY232, VAL234, LYS249, ILE251, ASN269, GLU273, LYS349, GLU351, ASN352, ASP368, GLY370
1OVE	P38 alpha	110.982	1	**H bonds:** ALA111
				**Interacting residues:** VAL30, TYR35, VAL38, ALA51, VAL52, LYS53, GLU71, LEU75, ILE84, LEU86, LEU104, THR106, HIS107, LEU108, MET109, GLY110, ASP112, ALA157, VAL158, LEU167, ASP168
1VKX	NF-Kβ	57.	3	**H bonds:** ARG33, ASN186, LYS218
				**Interacting residues:** ARG187, ALA188, PRO189, ALA192, GLU193, ASP217
3VVH	MEK1	66.5941	6	**H bonds**: GLY77, ASN78, LYS97, GLU144, MET146, SER194
				**Interacting residues**: LEU74, GLY75, ALA76, GLY79, GLY80, VAL81, VAL82, ALA95, VAL127, MET143, HIS145, GLN153, LYS192, ASN195, LEU197, CYS207, ASP208
2E9P	CHK1	84.1742	1	**H bonds:** MET42
				**Interacting residues:** ALA19, TYR20, LYS38, VAL40, ASP41, LYS43, ASN51, ILE52, GLU55, LEU82, GLY150, LEU151
2O21	Bcl-2	98.77	1	**H bonds:** PHE150
				**Interacting residues:** PHE101, TYR105, ASP108, PHE109, MET112, VAL130, GLU133, LEU134, GLY142, ARG143, VAL145, ALA146, GLU149, VAL153

## Discussion

CGs have been recognized as the oldest drugs and used for centuries to treat congestive heart diseases. Epidemiological reports recommended that cancer patients under treatment with digitalis showed less mortality rates compared to others, which reintroduced the attention toward the antiproliferative activities of CGs (14, 15). In the past 10 years, several *in vitro* and *in vivo* studies have been conducted, and many of the CGs or its derivatives are in clinical trials for cancer treatments (16). Several studies have reported that CGs like digoxin and digitoxin have reproducible anticancer effects. The results of this study also suggest a significant difference in cytotoxic concentration of strophanthidin compared to other CGs, which revealed that the underlying mechanism of apoptosis varies between commonly used anticancer drugs. For example, proscillaridin A, ouabain, and digoxin were shown to attenuate the proliferation by DNA topoisomerase inhibition activity, and oleandrin inhibits the growth of lung cancer cells to initiate apoptosis by upregulating the expression of DR4 (17). In the current study, we found that strophanthidin, one of the less-studied CGs, has anticancer activity in breast, lung, and liver cancer cell lines and contains no toxicity in non-malignant cell lines as well as PBMCs. It has been reported that CGs are toxic to a wide range of cell types, which can be a possible reason for their failures in clinical trials. We discovered the possible mechanism underlying the cell death induced by strophanthidin, which includes downregulation of proto-oncogenes, upregulation of TSGs, cell cycle arrest at the G2/M phase, apoptosis through expression changes in MAPK signaling, significant changes in the expression of PI3K/AKT/mTOR and Wnt/β-catenin signaling, and upregulation and downregulation of pro-apoptotic and antiapoptotic genes/proteins (BAX and Bcl-2), respectively. The whole summary of expressions of all the proteins and genes used in this study is mentioned in Supplementary Table 2. The protein–ligand interactions through the molecular docking approach showed the promising results of strophanthidin interacting with various proteins from different biochemical pathways.

Proto-oncogenes normally encode proteins that play a critical role in cell proliferation by participating in cellular pathways. Oncogenes arise from proto-oncogenes and are known to regulate multiple cellular signal transduction pathways (18). Oncogenes get activated by the mutations in proto-oncogenes which can change their structure and functions. Identification of oncogenes that show an important role in the instigation and expansion of cancers has raised novel targets and expanded the suite of novel anticancer drugs. Numerous molecules, new drugs, and monoclonal antibodies that act by directly affecting the activity of proto-oncogenes have been developed (19). Here, we have studied the expressions of three proto-oncogenes, namely, c-Fos, c-Myc, and c-Jun, and found their consistent upregulation in cancer cells, and inhibition of these genes can result in apoptosis of various cancer cells (20, 21). A significant downregulation in the gene expression was observed in the 24-h treatment with strophanthidin. Taken together, our results suggest that strophanthidin induces apoptosis through proto-oncogene dysregulation. As it is reported that proto-oncogenes serve as the downstream targets for several pathways (22), the results from the present study could enhance the importance of proto-oncogenes in cancer therapy.

An important mechanism for the cancer cell to survive is by deregulating the cell cycle. Mutated oncogenes and deactivated TSGs could raise the dependence of cancer cells in the G1 phase (23). Generally, in the cell cycle, G1 progression is controlled by cyclin D1, which assembles combinatorially with CDK6. The loss of CDK6 activity results in the arrest of the cell cycle (24). We have found that 24 h of strophanthidin treatment to MCF-7, A549, and HepG2 results in cell cycle arrest at the G2/M phase. Further, we checked the expressions of cyclin D1, CDK6, and checkpoint kinases (Chk1 and Chk2). Previous reports stated that the upregulation of Chk1, Chk2, and cyclin D1 promotes tumor cell growth (25). Therefore, downregulation or inhibition of Chk1, Chk2, cyclin D1, and CDK6 could be an innovative target for cancer treatment (26). It has been previously reported that CGs such as bufalin, glucoevatromonoside, digitoxin, and ouabain induce cell cycle arrest at the G2/M phase by deregulating the expression of polo-like kinase 1 (Plk1) expression in cancer cells (27, 28). Taken together, we have found a significant downregulation/inhibition of these genes compared to untreated cells. Overall, our results recommend that strophanthidin induces cell cycle arrest at the G2/M phase, and this effect was further confirmed by observing the downregulation of cyclin-dependent and checkpoint kinases.

Human bodies use numerous sophisticated mechanisms to safeguard against many diseases including cancer. These mechanisms recognize DNA mutations and induce either repair or death of faulty DNA or cell before it becomes oncogenic. Apoptosis is a programmed biological process that leads to cell death through various intrinsic and extrinsic pathways. Caspases are a family of cysteine endoproteases that regulates inflammation and apoptosis (29). In mammals, caspases 3, 6, 7, 8, and 9 are known to exhibit the main role in apoptosis. Mechanisms behind the caspase-mediated apoptosis start with the overexpression of the initiator caspase 9. Once the initiator caspase is activated, it regulates the downstream effector caspases like caspases 3, 6, and 9. Stimulated caspase 3 cleaves α-fodrin, PARP, lamin A, and DFF and thereby induces cell death. Overexpression of caspase 8 was observed in the strophanthidin-treated cells compared to untreated controls. It has been previously reported that CGs induce apoptosis through inhibition of death receptor 4 (17), which is a common activator for FADD and caspase 8. Activated caspase 8 is known to play a crucial role in death receptor signaling; hence, the current study delineates the importance of this compound in the cell death mechanism through death receptor signaling. However, MCF-7 cells are in general deficient of caspase 3 protein due to the partial deletion of CASP-3 between nucleotide positions 345 and 391, which creates the loss of function for this particular protein (30, 31). Nevertheless, apoptosis is even possible in the lack of caspase 3 with the help of caspase 7/6, which retains the same function as that of caspase 3 (32, 33). We have checked the protein expressions of initiator caspases 7 and 9 and effector caspases like caspase 3 and PARP and found the significant activation in effector caspases and PARP, which suggests that strophanthidin mediates apoptosis by the activation of caspases.

MAPK signaling is a complicated process involved in the pathogenesis of human disorders ranging from cancers and neurodegenerative diseases. The stimulation of MAPK cascades leads to disease development by initiating neuronal apoptosis (34). Inhibitors of ERK1/2, MEK, JNK, and p38 MAPK are considered to be potential drugs for many diseases including cancer (35). We have checked the expressions of genes/proteins involved in the MAPK pathway to cause apoptosis with strophanthidin treatment. It has been reported earlier that any changes in the MAPK cascade could lead to the uncontrolled growth of the cancer cells (36). Any compound that can inhibit the expression of any of the proteins in MAPK signaling can be used for treating cancer (37). So far, only a few compounds such as cobimetinib and selumetinib were reported to inhibit MEK1 (38). In this study, we have found the downregulation of MEK1, MAPK24, and p44. MEK1 is a dual-specificity protein kinase that will get activated by the phosphorylation of Raf, and also it can be activated by a variety of cytokines, membrane depolarization, and calcium influx (39). Among this, p44/ERK1/2 (which is activated in response to the MEK1/2 through the phosphorylation of Thr202/Tyr204 and Thr185/Tyr187) is the one that gets adversely regulated by the dual specificity of MEK inhibitors (40). It has been reported that the pathway crosstalk is necessary for several proteins from various pathways. For instance, Ras/Raf/MEK/ERK and Ras/PI3K/PTEN/Akt pathways interact with each other to regulate growth and tumorigenesis (41). In the current study, we have found an upregulation of Phospho-MEK1/2 (Ser217/Ser221), and this could be a consequence of the phosphorylation of PI3K as reported in the earlier studies (42, 43). The activation of AKT is controlled by a multistep process that involves PI3K (44), and this phosphorylated AKT inhibits the GSK3α activity by forming an autoinhibitory pseudosubstrate sequence; GSK3α in turn phosphorylates several upstream targets, mainly AKT, through the feedback mechanism (45). Along with that, PI3K also controls the expression of mTOR complex 1 that contains mTOR, Raptor, mLST8, PRAS40, and DEPTOR, which play crucial roles in apoptosis and other cellular events (46). Furthermore, phosphorylated MEK1 leads to phosphorylation of the regulatory Tyr and Thr residues of ERKs at the next tier of the cascade, thereby causing their activation to lead to cell death (47), which is also witnessed in the present study. Moreover, we have observed overexpression in the phosphorylation of SAPK/JNK by using Phospho-SAPK/JNK (Thr183/Tyr185), and this phosphorylation could lead to the activation of pro-apoptotic genes such as *BAX* (48), which is also identified in the present study. Along with that, p38 MAPK contributes to the cell cycle arrest and tumorigenesis. Depending on the cell types, p38 MAPK can arrest the cell cycle at G0, G1/S, and G2/M transitions and can control cytoskeleton remodeling (49). The activation of p38 MAPK depends on the upstream events such as caspase activations (50), which are also identified in the present study. Taken together, our findings are consistent with the literature that states that CGs inhibit the expression of MEK1/2 and ERK1/2 signaling to inhibit cancer cell proliferation and also inhibit the *HIV-1* gene in HIV infections (51, 52).

Differential cell cycle modulation by strophanthidin led to a G2/M arrest, as it showed downregulation of cyclin D1 in MCF-7, A549, and HepG2, but differential expression of p53 was observed as downregulation in MCF-7 and A549 but not in HepG2 cells. As the TSGs act as negative controllers of oncogenes and checkpoint kinases. Many TSGs have activity in both malignant and non-malignant cells. But very few TSGs are known to be regulated only in the case of cancer and remains active in normal cells (53). For example, p53 is one of the crucial TSGs which are known to be associated with 50% of human cancers. This will express only in the cancer cell, and it will be inactive in normal cells (54). The major function of TSGs includes apoptosis/programmed cell death. In the current study, we have checked the expressions of two TSGs, namely, p53 and PTEN. Under normal conditions, p53 and PTEN express at a very low level unless the cells are activated by a variety of stress or DNA damage. The response to cellular stress and DNA damage results in the upregulation of TSGs (55). We have observed the DNA damage by comet assay in 24-h treatment with strophanthidin and the overexpression of PTEN. In contrast with the earlier results, we have identified that unlike glucoevatromonoside, strophanthidin also showed a differential expression in certain proteins, mainly p53, which leads to the cell cycle arrest at the G2/M phase (27). The obtained results in the present study were correlated with the findings of Wang et al. (28), showing CGs (digoxin and ouabain) also induce apoptosis independently of their p53 status (56). Several reports have suggested that PI3K/AKT/mTOR signaling contributes a central role between cell survival, apoptosis, and autophagy inhibition (57, 58). PI3K is known to promote cancer cell growth by its upregulation in the tumor/tumor cells and its role in cell survival, neovascularization, and proliferation. Overexpression of PI3K is reported in malignant cells, and the inhibitors of this could lead to the discovery of novel drug targets (59, 60). The activation of mTOR is also dependent on mitogen signaling through the PI3K/AKT/mTOR signaling through the pathway crosstalk mechanism (61). mTOR is a downstream regulator of the PI3K/AKT/mTOR pathway and is a known target for cancer therapy, and mTOR inhibition leads to the blockade of downstream pathways, controls the cell cycle, and ultimately leads to cell cycle arrest (62). This inhibition of mTOR can also diminish the chances of Wnt signaling by suppressing/knocking out effector proteins such as LC3 and Beclin 1, and it further raises Wnt transformed transcriptional movement, which suggests that Wnt signaling also plays a role in the inhibition of autophagy (63, 64). In the current study, we have found a significant downregulation of mTOR, PI3K, p62, and Beclin 1, which can effectively cause autophagy and could further induce apoptosis due to nutritional deficiency upon the stress created by strophanthidin. A similar result was observed by Hossan et al. (15), suggesting that CGs such as cerberin act through PI3K/AKT/mTOR signaling to inhibit autophagy and to induce apoptosis in cancer cells. Our results in the current study not only enrich the anticancer potentiality of strophanthidin but also give novel insights into the mechanism of the cytotoxic effect on PI3K/AKT/mTOR signaling for improved cancer treatment.

The Wnt/β-catenin pathway is implicated in several cellular functions ranging from embryonic development to cancer, cell proliferation, cell cycle, and cell death (65, 66). The Wnt/β-catenin pathway promotes cell death in cancer cells by deregulating (67), while activation leads to inhibition of apoptosis (68). Inhibition of GSK3α leads to the suppression of β-catenin, which further leads to the changes in the expression of cyclin-dependent kinase (cyclin D1) and proto-oncogene encoding protein (c-Myc). We have observed the inhibition of GSK3α and β-catenin by the activation of a destructive complex and further enhancing the deregulation of c-Myc and cyclin D1, stimulating cell death. In the current study, our results confirm strophanthidin's importance in the inhibition of Wnt/β-catenin signaling, and this activity was correlated with earlier reports suggesting that bufalin inhibits Wnt/β-catenin signaling in gastric cancer cells to induce apoptosis (69). However, this is the first report to show the role of strophanthidin in Wnt/β-catenin signaling and its downstream target genes.

The anticancer activity of strophanthidin has been revealed in the current study. However, the exact binding modes and molecular interactions have not been elucidated for most of the protein targets. In this study, we initiated an *in silico* approach to identifying the novel interacting residues of these protein targets which mediate the binding of strophanthidin for the first time. Along with that, the exact mode of inhibition among the multiple modes possible is also shown. Moreover, the present study used docking approaches to identify interacting residues of many important proteins like STAT3, p38MAPK, NF-κB, Chk1, Bcl-2, Chk2, MEK1, PARP-1, and cyclin D1 for the first time. Strophanthidin inhibits the target molecules by influencing their functionally important residues and thereby results in function loss. Strophanthidin–protein architecture is anticipated to guide scientists by providing an appropriate model for a multitarget drug structure and by providing structural details on the inhibitory mechanism. As per our knowledge, this is the first attempt to identify the molecular interactions in CGs with various crucial proteins from different pathways.

## Conclusion

**Figure 10:**
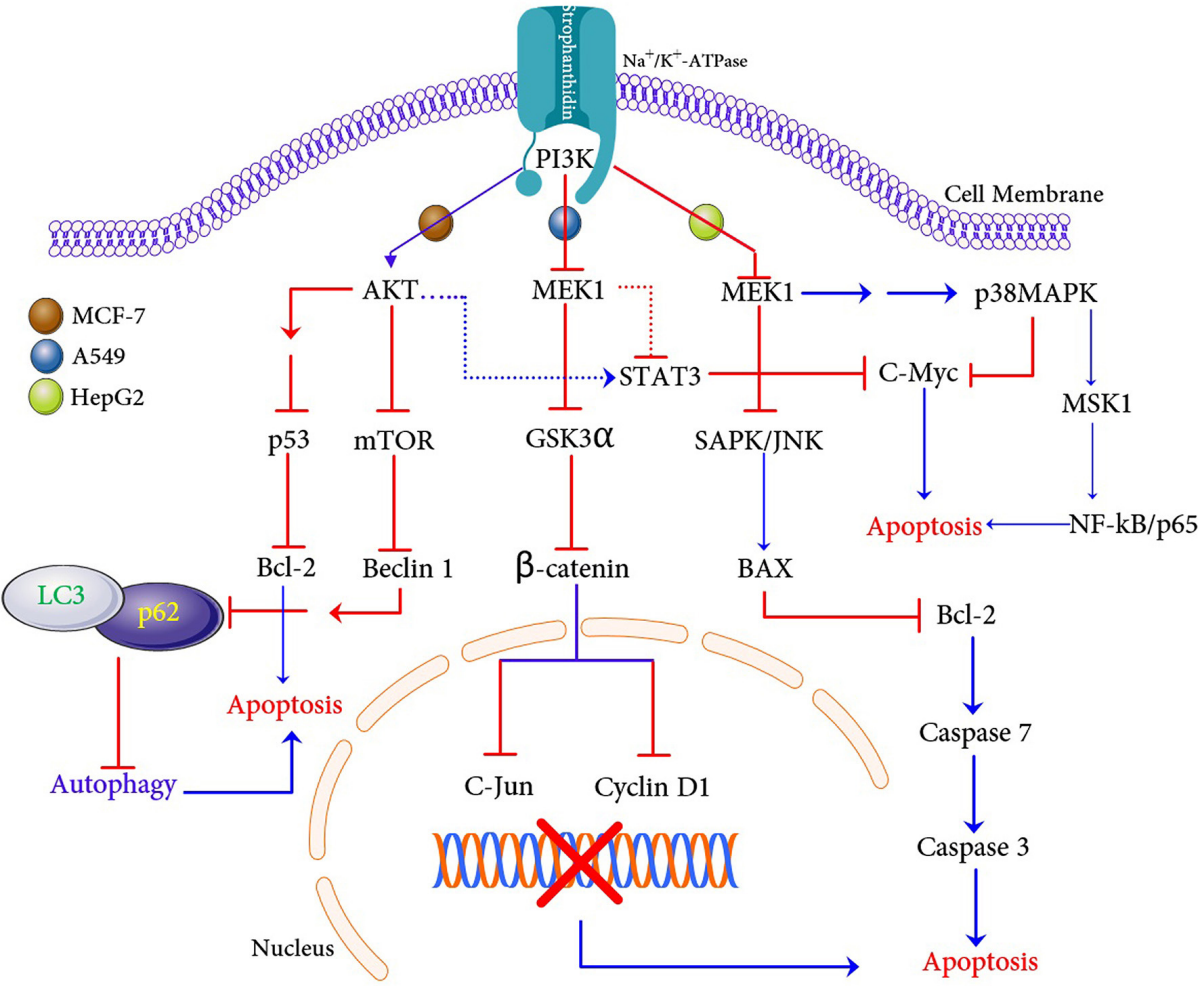
Proposed apoptosis and autophagic cell death mechanism induction with strophanthidin in MCF-7, A549, and HepG2 cancer cells.

In conclusion, our *in vitro* and *in silico* experiments demonstrate that a natural compound strophanthidin has a potent anticancer effect on breast, lung, and liver cancer cells. We have demonstrated the possible hypothetical mechanism of apoptosis in these three cancer cells (Figure 10). Strophanthidin hinders the expression of PI3K in MCF-7 cells, which is a key protein for PI3K/AKT/mTOR signaling. This inhibition leads to further activation of AKT, and the inhibition of mTOR and also shows multistep inhibition of p53, which in turn leads to the deregulation of anti-apoptotic protein (Bcl-2) and apoptosis. Suppression of mTOR leads to the downregulation of Beclin 1 and stimulates the inhibition of LC3 and p62 complex to inhibit autophagy. Inhibition of MEK1 was identified in A549 cells due to the downregulation of PI3K and plays a crucial role by inhibiting Gsk3α and β-catenin, which can directly target c-Myc and cyclin D1 to influence apoptosis. Dysregulated MEK1 targets p38MAPK and inhibits the expression of STAT3 and c-Myc to initiate apoptosis. A549 cells exhibit solid autophagic flux compatible with their proliferation rate, and strophanthidin inhibits the expression of LC3 and p62 complex through MEK1 inhibition; hence, it seems that this compound can inhibit autophagy. The inhibition of autophagy exacerbates apoptosis, which is a critical mechanism for a cancer cell to maintain the cellular energy and nutritional homeostasis. MEK1 also activated the expression of BAX by multistep stimulation, which could be an important factor for the activation of initiator caspase 7 and further leads to the expression of caspase 3 and causes apoptosis through death receptor signaling and caspase-mediated apoptosis as witnessed in HepG2 cells. Moreover, strophanthidin exhibited a differential expression that shows the p53-dependent and p53-independent apoptosis in cancer cells. To the best of our knowledge, this is the first study to identify the changes in various biochemical apoptotic signal transduction pathways like MAPK signaling, PI3K/AKT/mTOR, and Wnt/β-catenin pathways to initiate apoptosis. Conclusively, this study revealed for the first time that strophanthidin induced apoptosis by the attenuation of multiple biochemical signaling pathways and by arresting cell cycle at the G2/M phase through p53-dependent and p53-independent mechanisms.

## Data Availability Statement

All datasets generated for this study are included in the article/Supplementary Material.

## Author Contributions

RK and DR: conceived and designed the experiments. RK: analyzed the data and contributed reagents, materials, and analysis *in silico* study tools. DR, RK, and PG: wrote the paper/made language corrections.

### Conflict of Interest

The authors declare that the research was conducted in the absence of any commercial or financial relationships that could be construed as a potential conflict of interest.
