# Supplementary material for: Strophanthidin Attenuates MAPK, PI3K/AKT/mTOR, and Wnt/β-Catenin Signaling Pathways in Human Cancers
Source: Front Oncol. 2020 Jan 17;9:1469. doi: 10.3389/fonc.2019.01469 (PMC6978703; doi:10.3389/fonc.2019.01469)
Supplement: Supplementary file 1 [file Data_Sheet_1.PDF]

Supplementary Data

Strophanthidin attenuates MAPK, PI3K/AKT/mTOR and Wnt/β-catenin signaling pathways in Human Cancers

Dhanasekhar Reddy[1], Preetam Ghosh[2] and Ranjith Kumavath[1*]

[1]Department of Genomic Science, School of Biological Sciences, Central University of Kerala, Tejaswini Hills, Periya(P.O) Kasaragod, Kerala-671316, India.

[2]Department of Computer Science, Virginia Commonwealth University, Virginia 23284, USA.

***Corresponding author:** Dr. Ranjith Kumavath Ph.D., FSAB.
Email: RNKumavath@gmail.com; RNKumavath@cukerala.edu.in Tel**.:** +91-8547648620

Gene	Forward primer	Reverse primer
BCL2	TTGTGGCCTTCTTTGAGTTCGGTG	GGTGCCGGTTCAGGTACTCAGTCA
BAX	CCTGTGCACCAAGGTGCCGGAACT	CCACCCTGGTCTTGGATCCAGCCC
CDK6	GGATAAAGTTCCAGAGCCTGGAG	GCGATGCACTACTCGGTGTGAA
CHK1	TTGGCTTCCTGCCACATGAT	TTGCAGTTTGCAGGACAGGA
CHK2	AGTGGTGGGGAATAAACGCC	TCTGGCTTTAAGTCACGGTGTA
P53	CAGCACATGACGGAGGTTGT	TCATCCAAATACTCCACACGC
MEK1	TGAGAGCGACGGTTCTCTACT	CACAATCAGAGTGTCCTGTTGTT
P44	ACTATGTCCGAAGCAAGGATTTC	CGCCCACTGATAATCTCTGGAG
P38 α	AACCTGTCTCCAGTGGGCTCT	CGTAACCCCGTTTTTGTGTCA
SAPK/JNK	GGGTATGCCCAAGAGGACAGA	GTGTTGGAAAAGTGCGCTGG
NF-Kβ -P65	GTCAAAAACGCCACCTCTCAA	CTCGCATGGAATTTGGAACCG
AKT	CCTCCACGACATCGCACTG	TCACAAAGAGCCCTCCATTATCA
STAT3	CAGCAGCTTGACACACGGTA	AAACACCAAAGTGGCATGTGA
JAK	GCCAACGAGGATCTTCGAGC	CTTCTCGCGTTCCACTTTGC
P62	GCACCCCAATGTGATCTGC	CGCTACACAAGTCGTAGTCTGG
mTOR	ATGCAGCTGTCCTGGTTCTC	AATCAGACAGGCACGAAGGG
Pi3K	CCACGACCATCATCAGGTGAA	CCTCACGGAGGCATTCTAAAGT
β-Catenin	AGCTTCCAGACACGCTATCAT	CGGTACAACGAGCTGTTTCTAC
c-MYC	ATGGCCCATTACAAAGCCG	TTTCTGGAGTAGCAGCTCCTAA
CYCLIN D1	GCTGCGAAGTGGAAACCATC	CCTCCTTCTGCACACATTTGAA
C-JUN	TGACTGCAAAGATGGAAACG	CAGGGTCATGCTCTGTTTCA
C-FOS	AAGGGAAAGGAATAAGATGGCT	GCAAAGCAGACTTCTCATCT
LC3	GGAGAATCCGAAGGGAAAG	TTGAGCTGTAAGCGCCTTCTA
Beclin 1	CTGGTAGAAGATAAAACCCGGTG	AGGTAGAGCGTGGACTATCCG
Sestrin 1	TGCTTTGGGCCGTTTGGATAA	TGTAGTGACGATAATGTAGGGGT
MAPK24 (MKK4)	GACGAGGAGCTTATGGTTCTGT	TTTTCATCCACTGTTGACCGAA
PTEN	AGGGACGAACTGGTGTAATGA	CTGGTCCTTACTTCCCCATAGAA
Msk1	CAACAATCGTTCAAAAGGCCAA	CGACTGCCTAATGTGTTCCAG
Gsk3A	GTGCCCGAGACAGTGTACC	ACACCTTGACATAGAGGATAGGG
GAPDH	AACGGGAAGCTTGTCATCAATGGAAA	GCATCAGCAGAGGGGGCAGAG

Supplementary Table S1: List of primers used in this study. All the primers were obtained from PrimerBank. Primers were chosen for cell cycle regulating genes, proto-oncogenes, tumor suppressor genes and genes from various pathways such as MAPK signaling, NF-Kβ signalling, AKT-Mtor-Pi3K pathway and Wnt/β-Catenin signalling.

S. no	Gene/protein	Expressions (Up/Down) in Cell Lines		
		MCF-7	A549	HepG2
1.	*C-Fos*	↓	↓	↓
2.	*C-Myc*	↓	↓	↓
3.	*C-Jun*	↓	↓	↓
4.	*Chk1*	↓	↓	↓
5.	*Chk2*	↓	↓	↓
6.	*Cdk6*	↓	↓	↓
7.	*Cyclin D1*	↓	↓	↓
8.	*Jak*	↓	↓	↓
9.	*PI3K*	↓	↓	↓
10.	*mTOR*	↓	↓	↓
11.	*MEK1*	↓	↓	↓
12.	*p62*	↓	↓	↓
13.	*Beclin*	↓	↓	↓
14.	*Sestrin*	↑	↓	↓
15.	LC3	↓	↓	↓
16.	*Gsk3α*	↓	↓	↓
17.	*β-catenin*	↓	↓	↓
18.	*Bcl-2*	↓	↓	↓
19.	*AKT*	↑	↑	↑
20.	*p38MAPK*	↑	↑	↑
21.	*BAX*	↑	↑	↑
22.	*NF-kβ*	↑	↑	↑
23.	*MAPK24*	↑	↓	↓
24.	*p44*	↑	↓	↓
25.	*STAT3*	↑	↓	↑
26.	*Msk1*	↑	↑	↓
27.	*p53*	↓	↓	↑
28.	*PTEN*	↓	↑	↑
29.	*SAPK/JNK*	↓	↑	↑

Supplementary table S2: Summary of identified gene/protein expressions in this study upon Strophanthidin treatment.

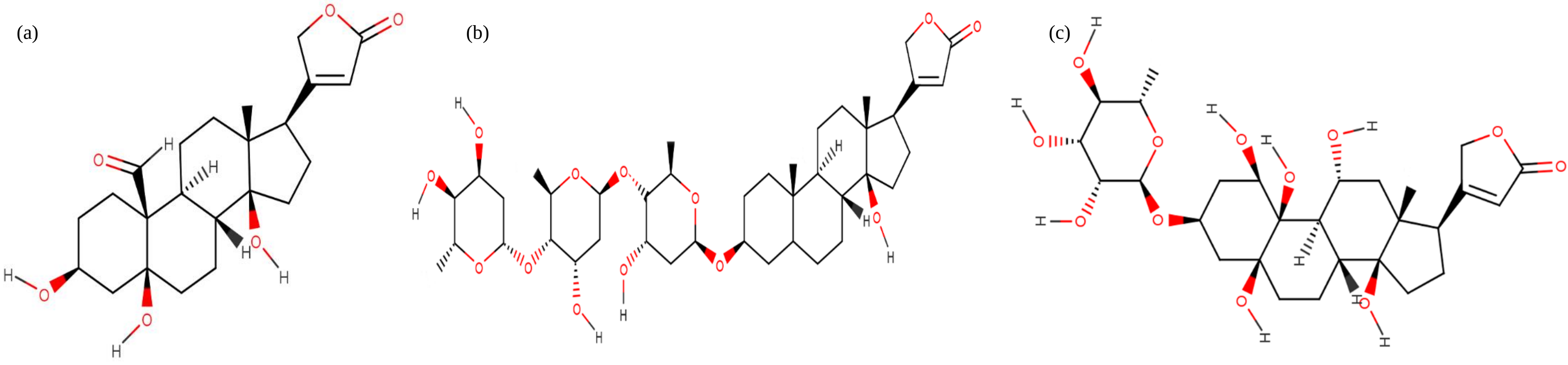

(a) **Strophanthidin**

(b) **Digitoxin**

(c) **Ouabain**

Supplementary Fig. 1: Structural comparison of Strophanthidin (a) with Digitoxin (b) and Ouabain(c). All the chemical structures of compounds were drawn by using Chemdraw.

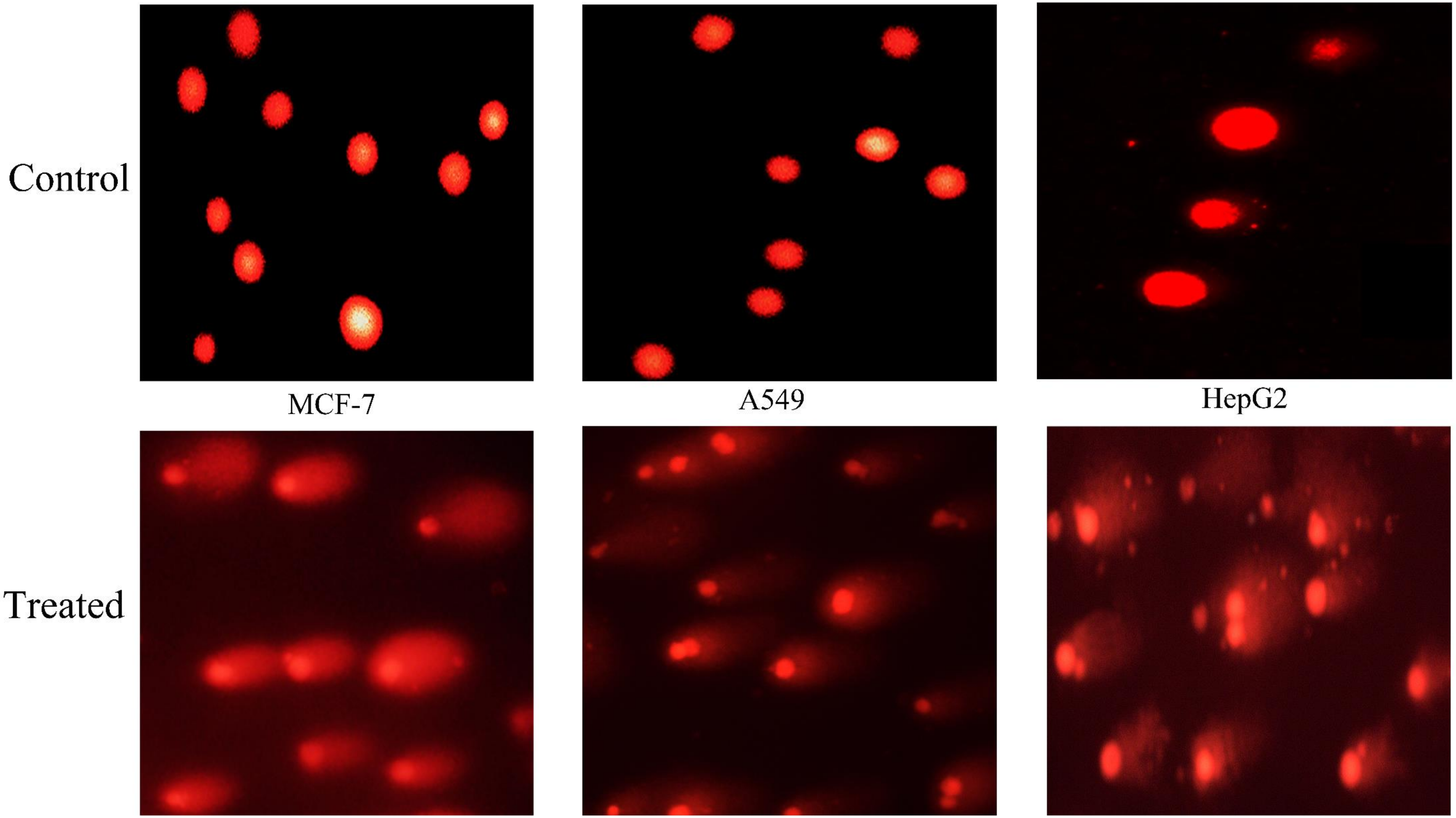

Supplementary Fig. 2: Comet assay showing DNA damage in Control and treatments in MCF-7, A549 and HepG2 cells.

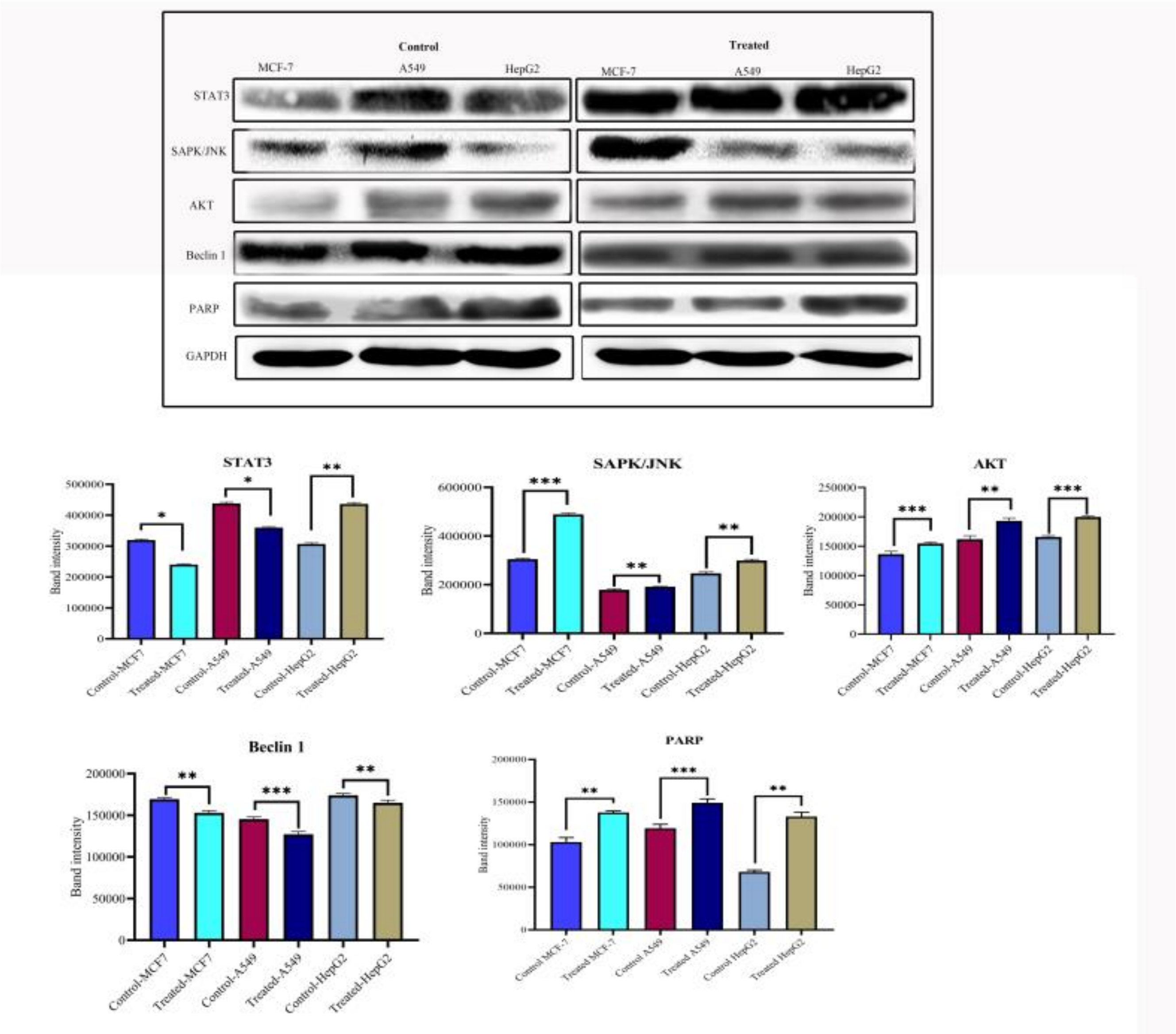

Supplementary Fig 3: Western blot expressions of target proteins with Strophanthidin treatment. Expression of several key proteins from various signaling pathways such as STAT3, SAPK/JNK, AKT, Beclin 1 and PARP. Blots were compared with that of GAPDH expression to compare equal loading of samples.

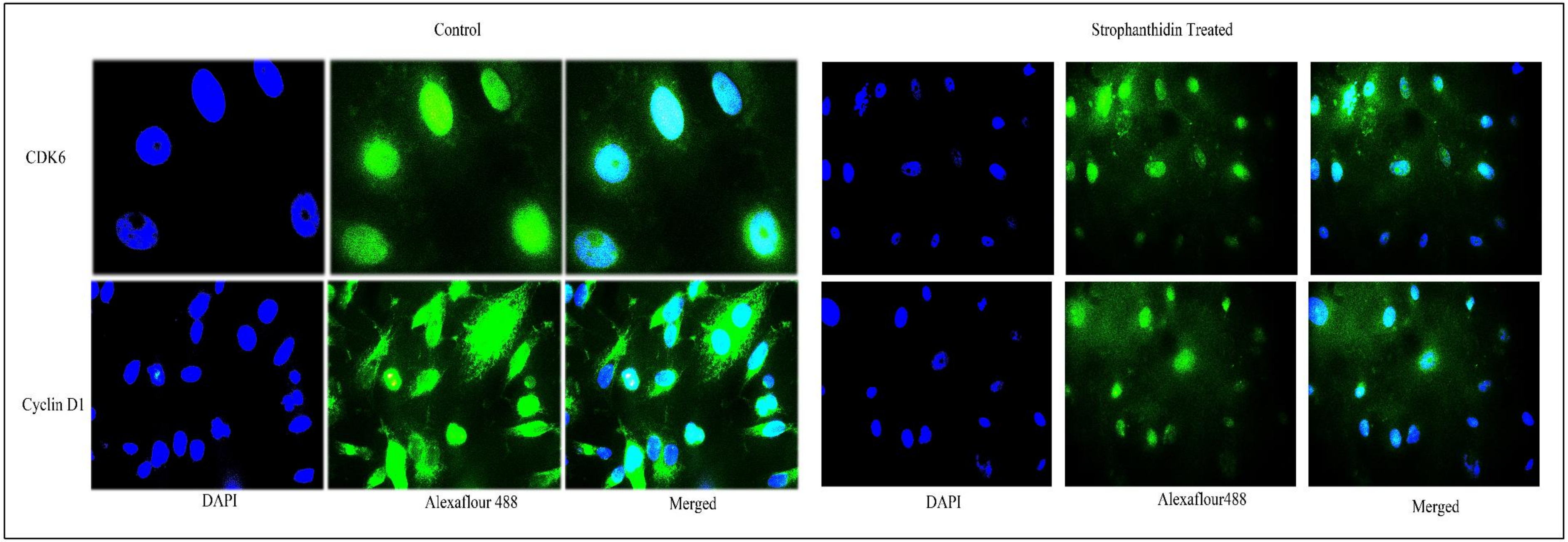

Supplementary Fig. 4A: Immunofluorescence imaging for the analysis of protein localisations of CDK6 and Cyclin D1 in Strophanthidin induced MCF-7 cells.

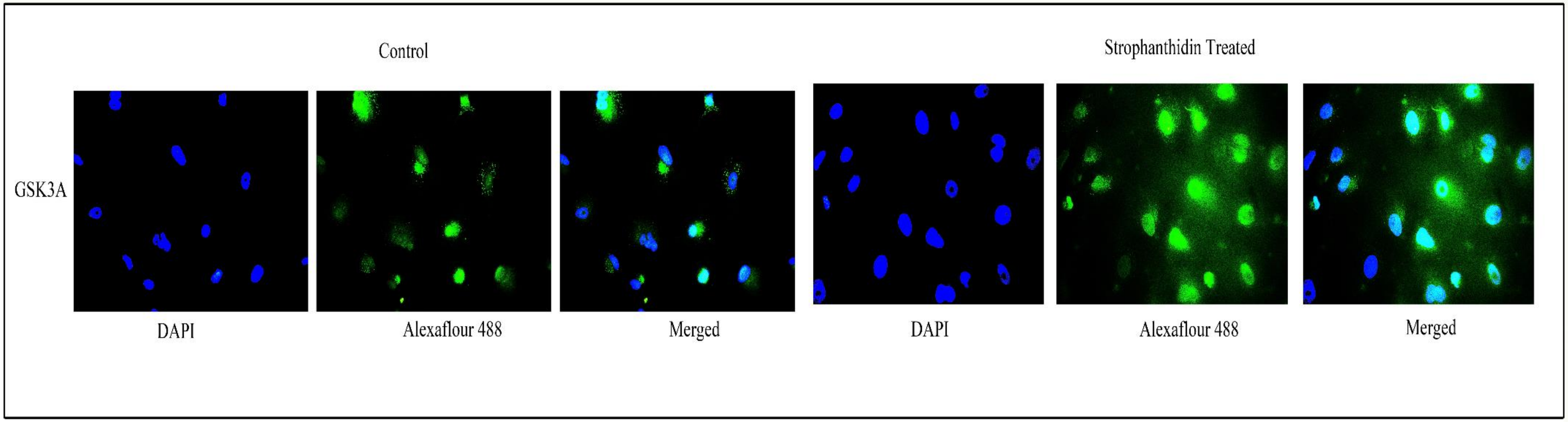

Supplementary Fig. 4B: Immunofluorescence imaging for the analysis of protein localisation of Gsk3α in Strophanthidin induced MCF-7 cells.

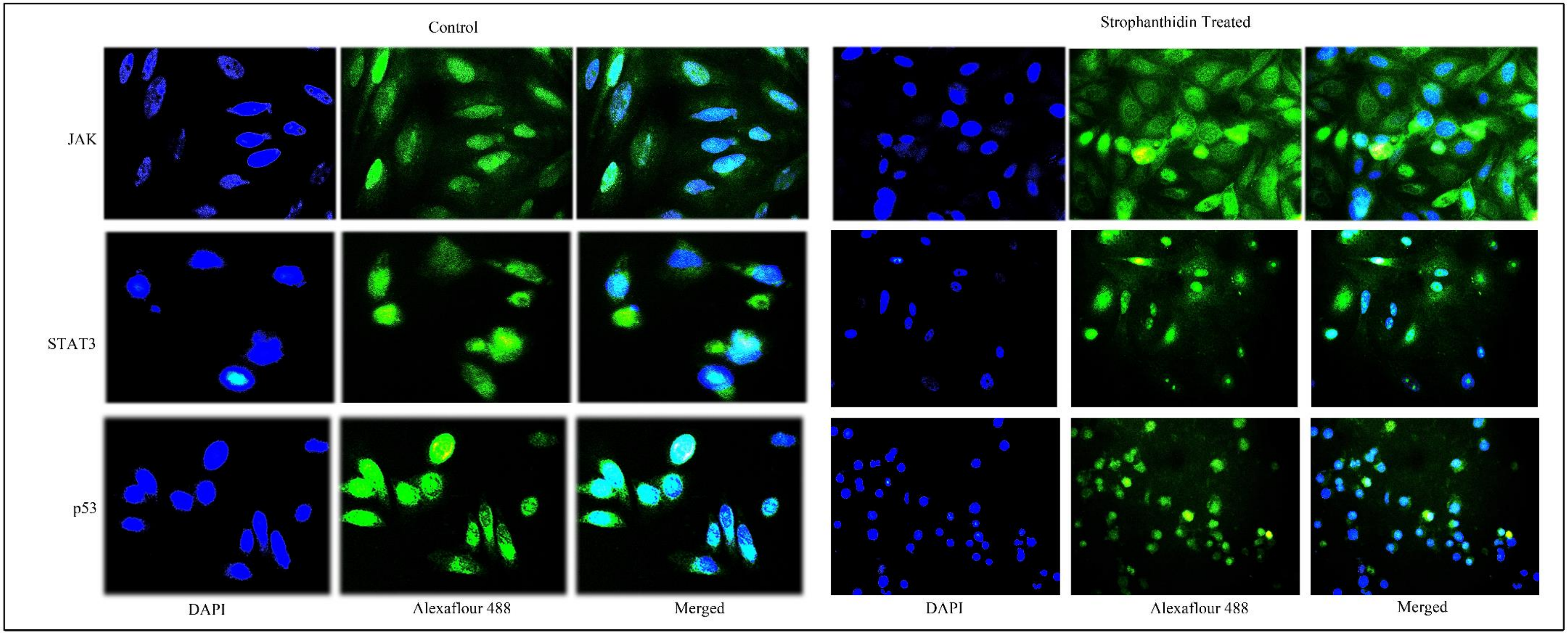

Supplementary Fig. 4C: Immunofluorescence imaging for the analysis of protein localisations of JAK, STAT3 and p53 in Strophanthidin induced MCF-7 cells.

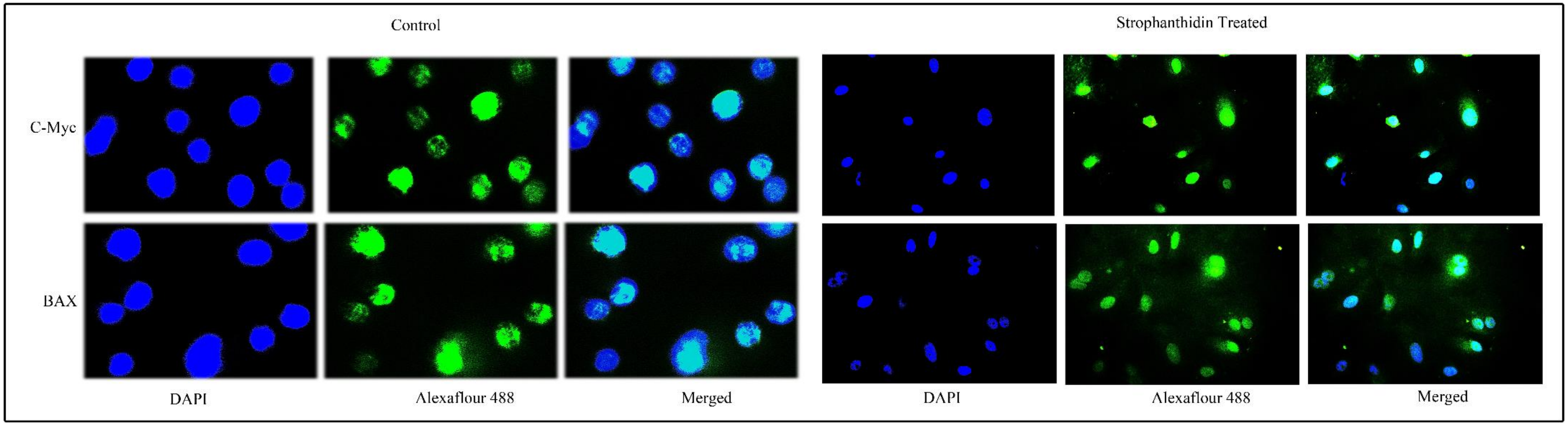

Supplementary Fig. 4D: Immunofluorescence imaging for the analysis of protein localisations in c-Myc and BAX in Strophanthidin induced MCF-7 cells.

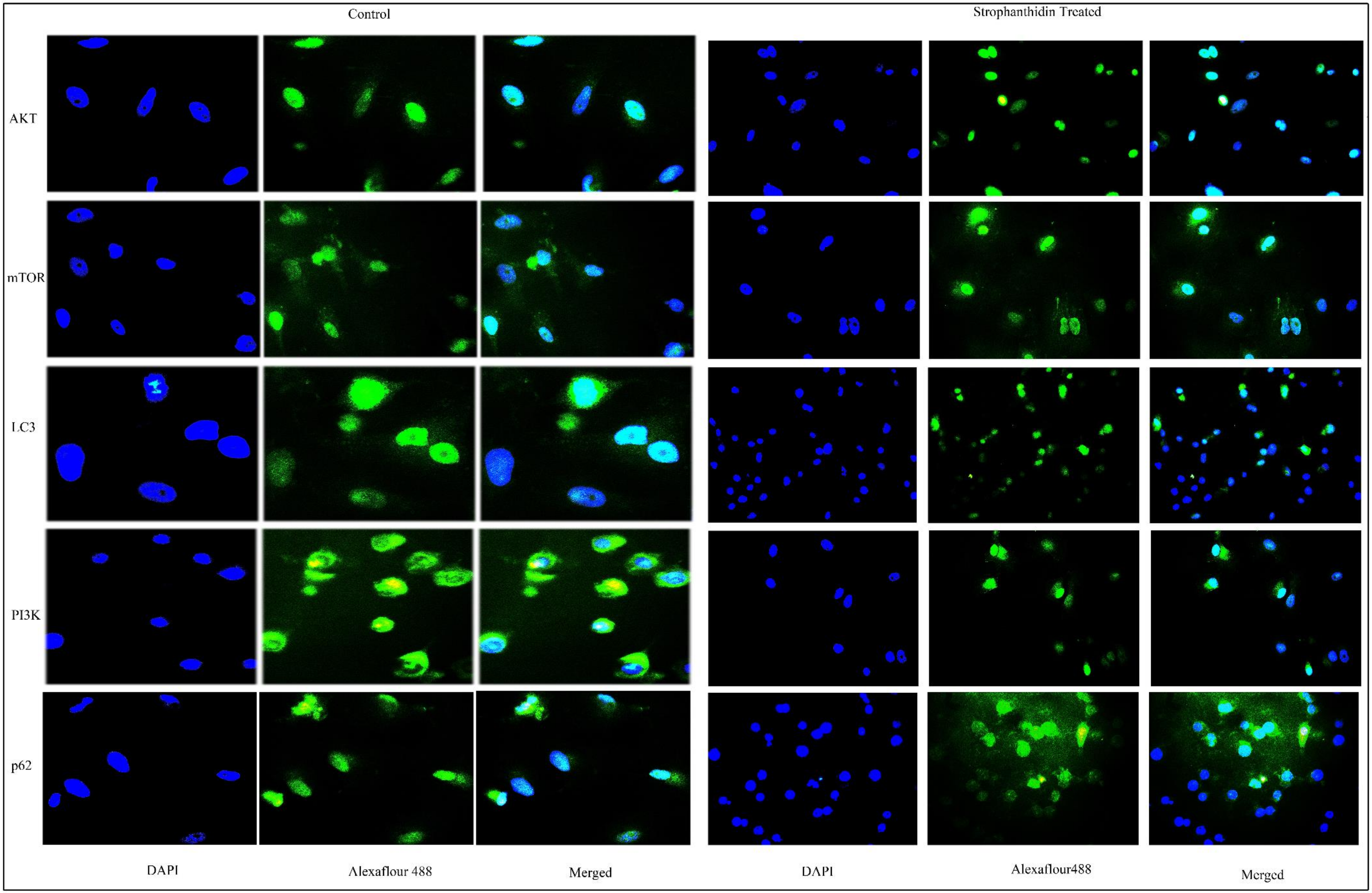

Supplementary Fig. 4E: Immunofluorescence imaging for the analysis of protein localisations in AKT, mTOR, LC3, PI3K and p62 in Strophanthidin induced MCF-7 cells.

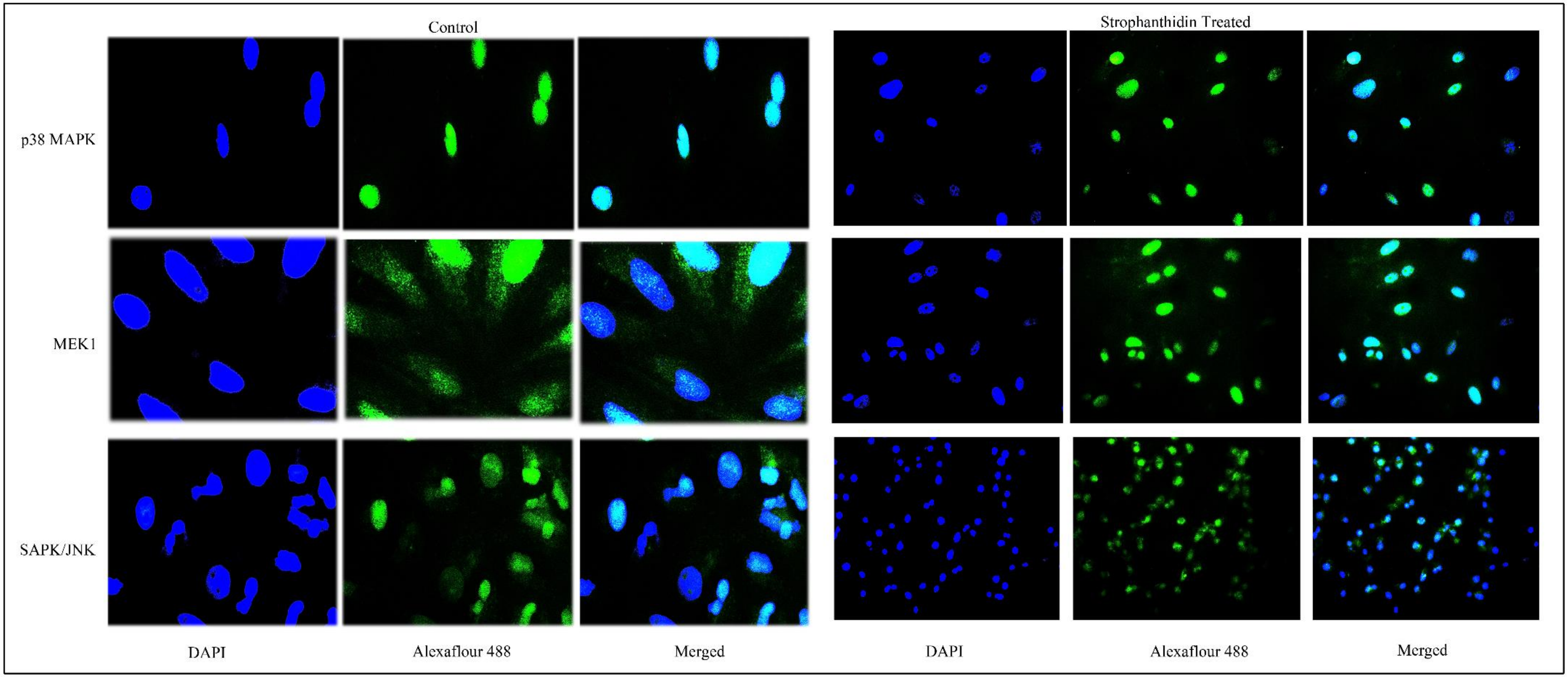

Supplementary Fig. 4F: Immunofluorescence imaging for the analysis of protein localisations of p38MAPK, MEK1and SAPK/JNK in Strophanthidin induced MCF-7 cells.

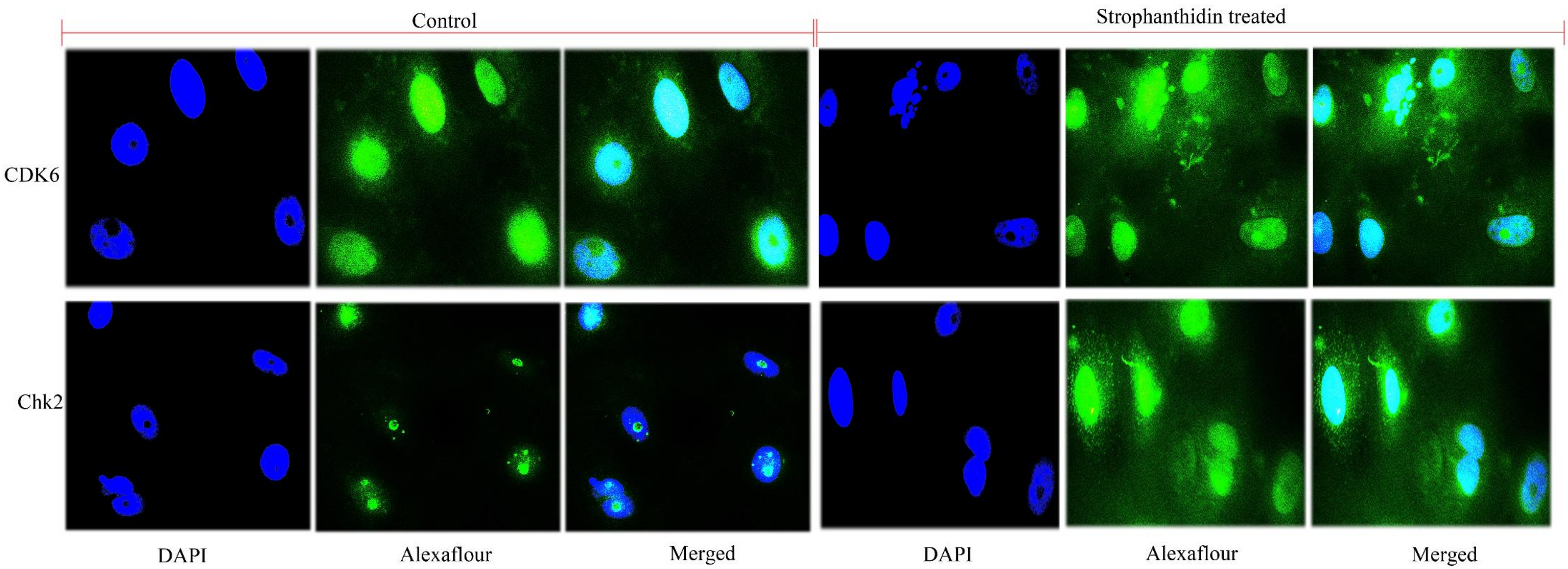

Supplementary Fig. 4G: Immunofluorescence imaging for the analysis of protein localisations of CDK6 and Chk2 in Strophanthidin induced MCF-7 cells.

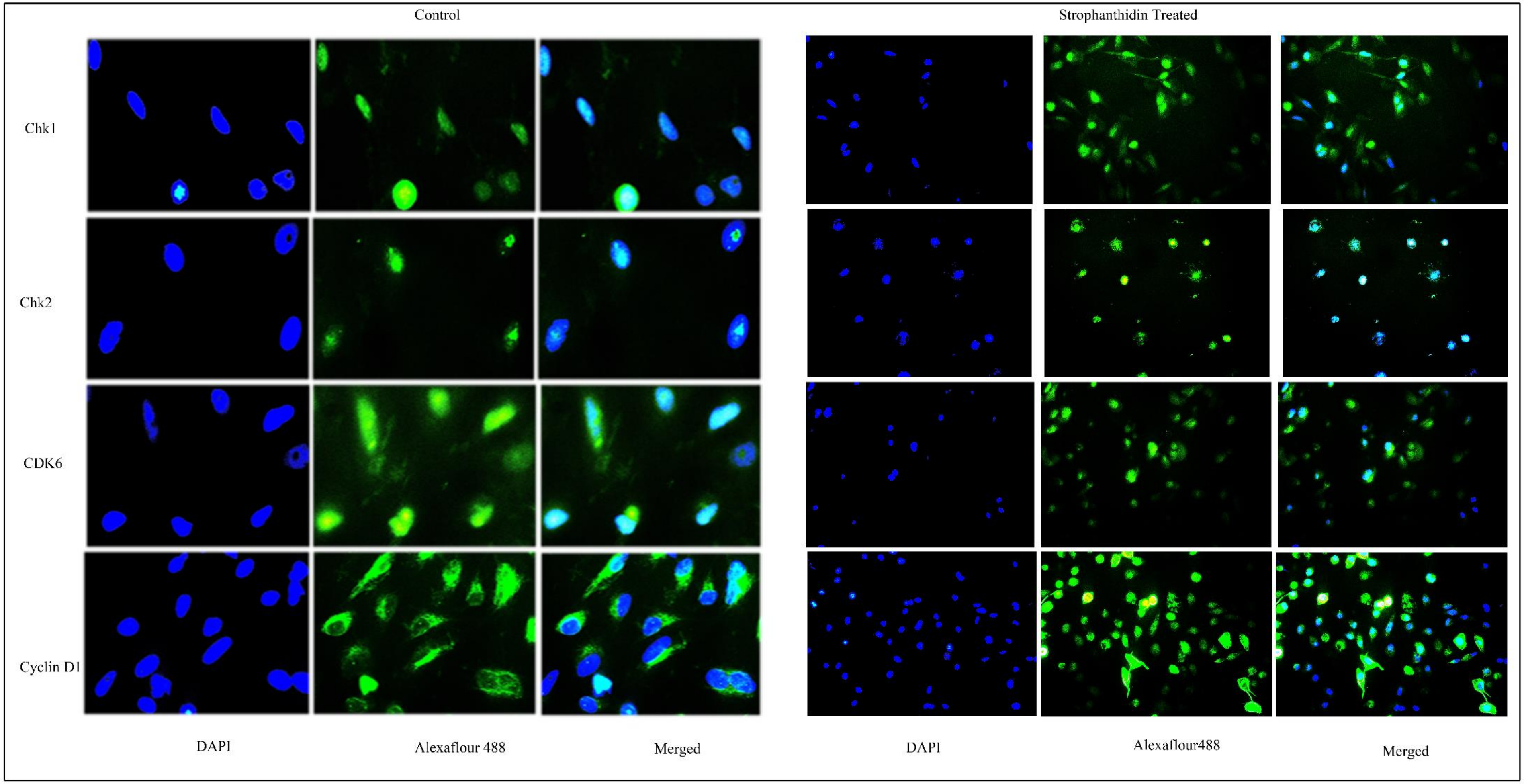

Supplementary Fig. 5A: Immunofluorescence imaging for the analysis of protein localisations of Chk1, Chk2, CDK6 and Cyclin D1 in Strophanthidin induced A549 cells.

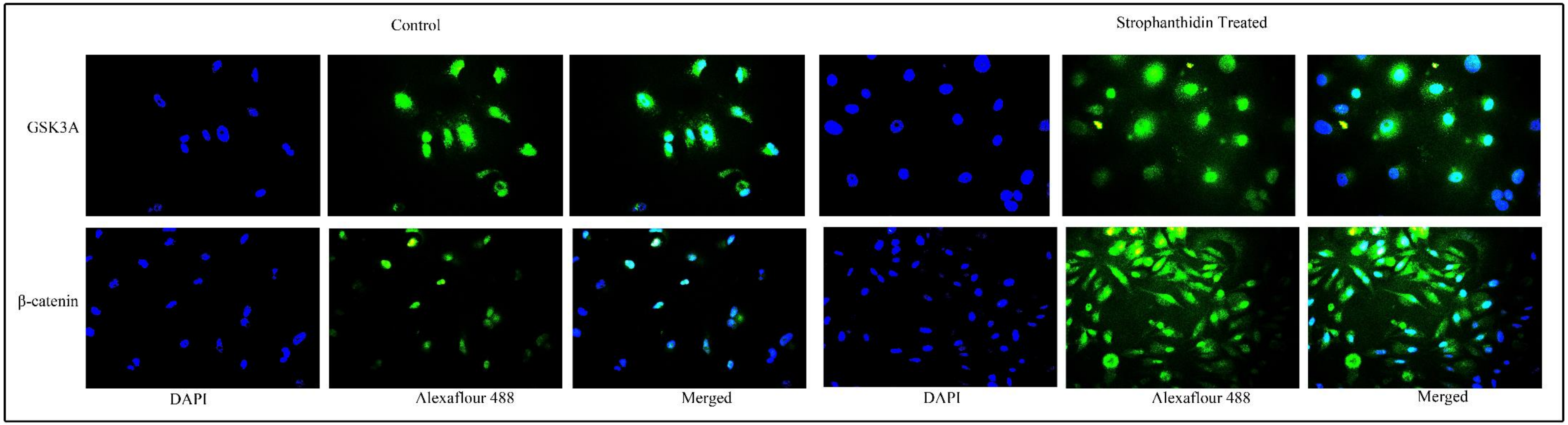

Supplementary Fig. 5B: Immunofluorescence imaging for the analysis of protein localisation of Gsk3α and β-catenin in Strophanthidin induced A549 cells.

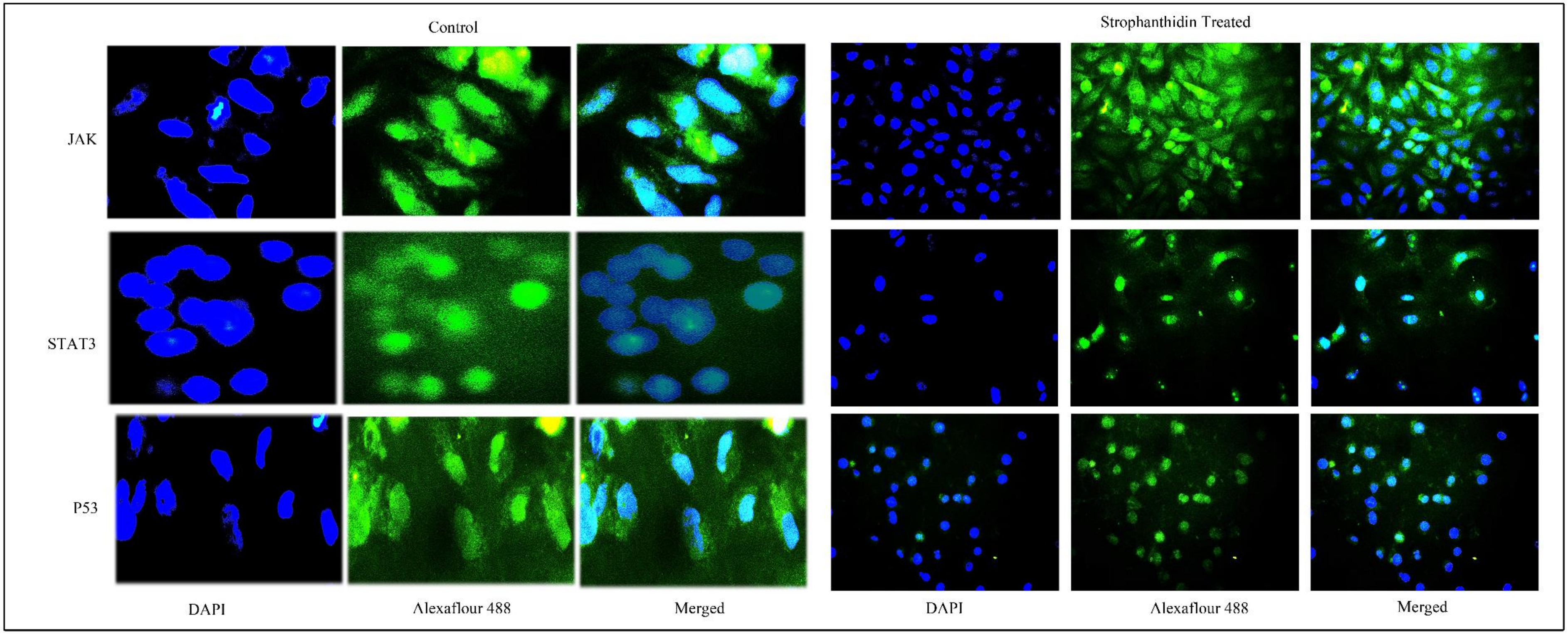

Supplementary Fig. 5C: Immunofluorescence imaging for the analysis of protein localisations of JAK, STAT3 and p53 in Strophanthidin induced A549 cells.

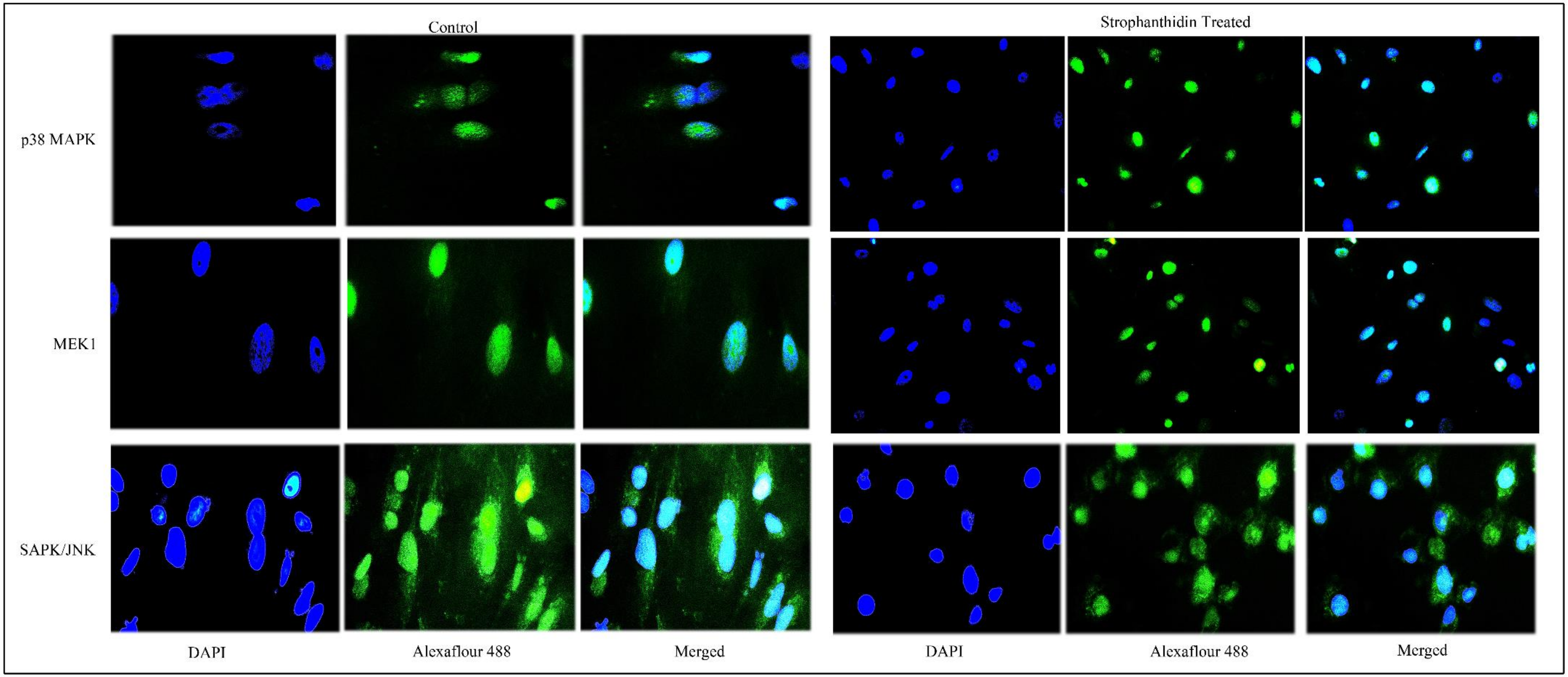

Supplementary Fig. 5D: Immunofluorescence imaging for the analysis of protein localisations of p38MAPK, MEK1and SAPK/JNK in Strophanthidin induced A549 cells.

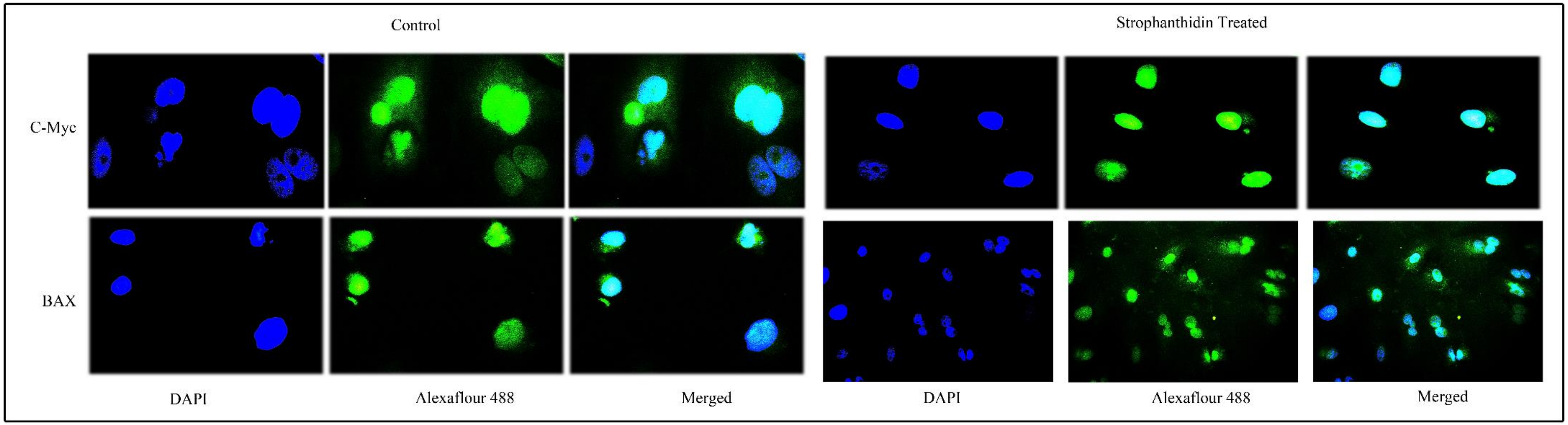

Supplementary Fig. 5E: Immunofluorescence imaging for the analysis of protein localisations in c-Myc and BAX in Strophanthidin induced A549 cells.

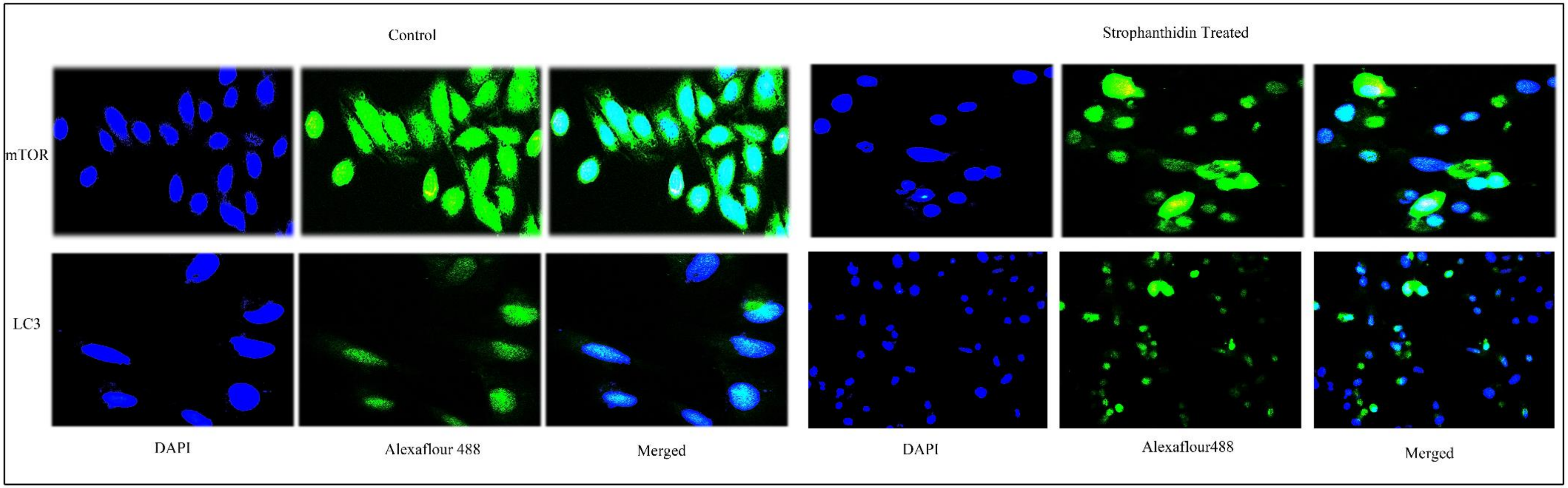

Supplementary Fig. 5F: Immunofluorescence imaging for the analysis of protein localisations in mTOR, and LC3 in Strophanthidin induced A549 cells.

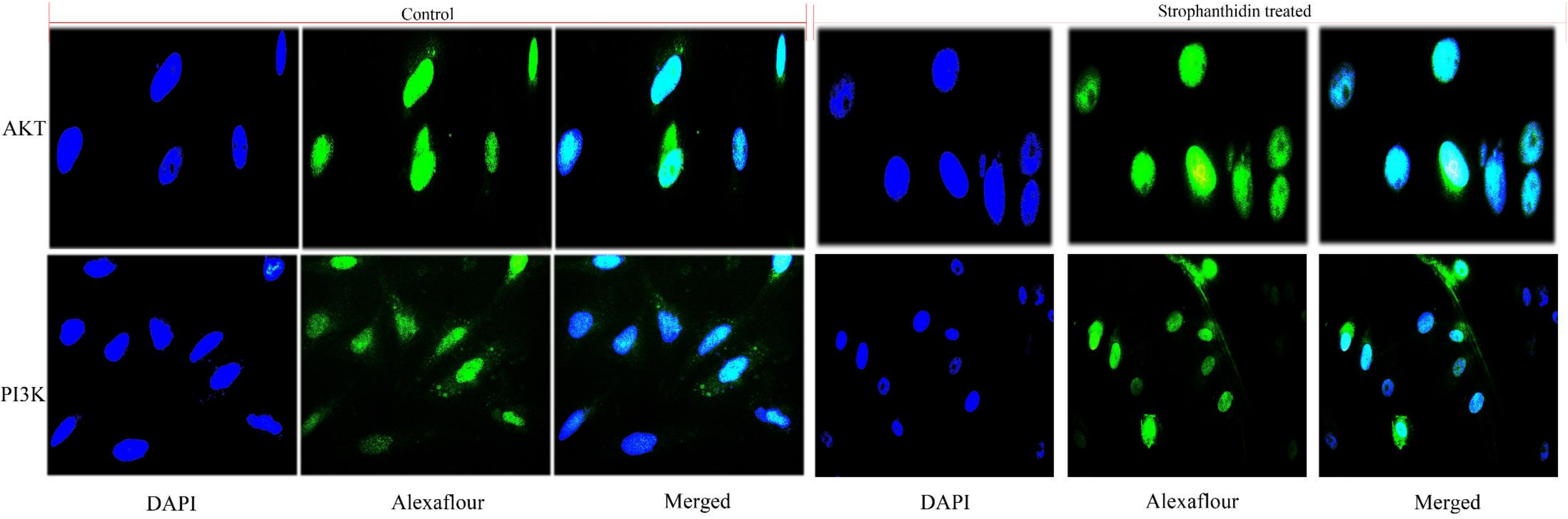

Supplementary Fig. 5G: Immunofluorescence imaging for the analysis of protein localisations in AKT, and PI3K in Strophanthidin induced A549 cells.

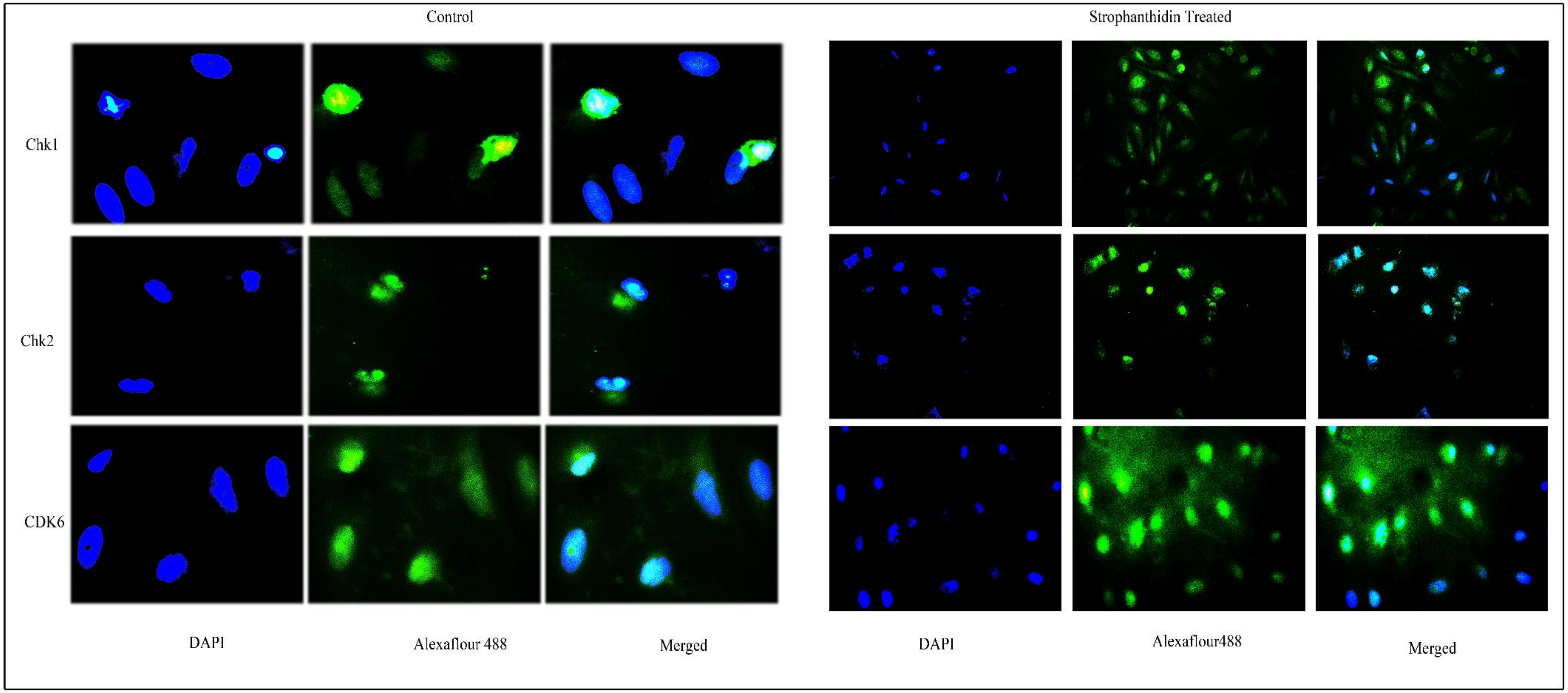

Supplementary Fig. 6A: Immunofluorescence imaging for the analysis of protein localisations of Chk1, Chk2, and CDK6 in Strophanthidin induced HepG2 cells.

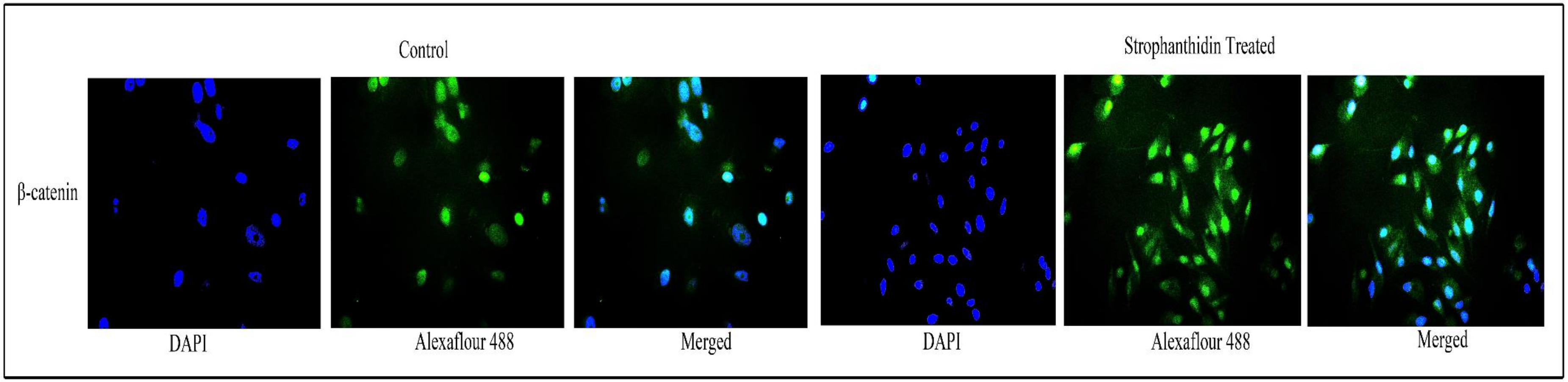

Supplementary Fig. 6B: Immunofluorescence imaging for the analysis of protein localisation of β-catenin in Strophanthidin induced HepG2 cells.

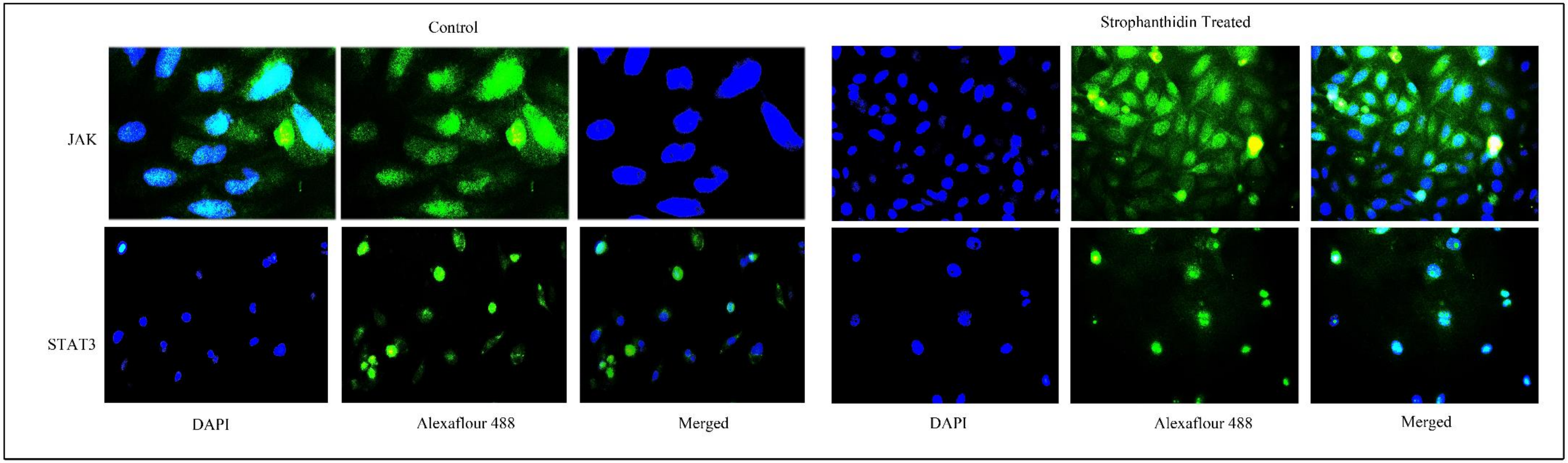

Supplementary Fig. 6C: Immunofluorescence imaging for the analysis of protein localisations of JAK, and STAT3 in Strophanthidin induced HepG2 cells.

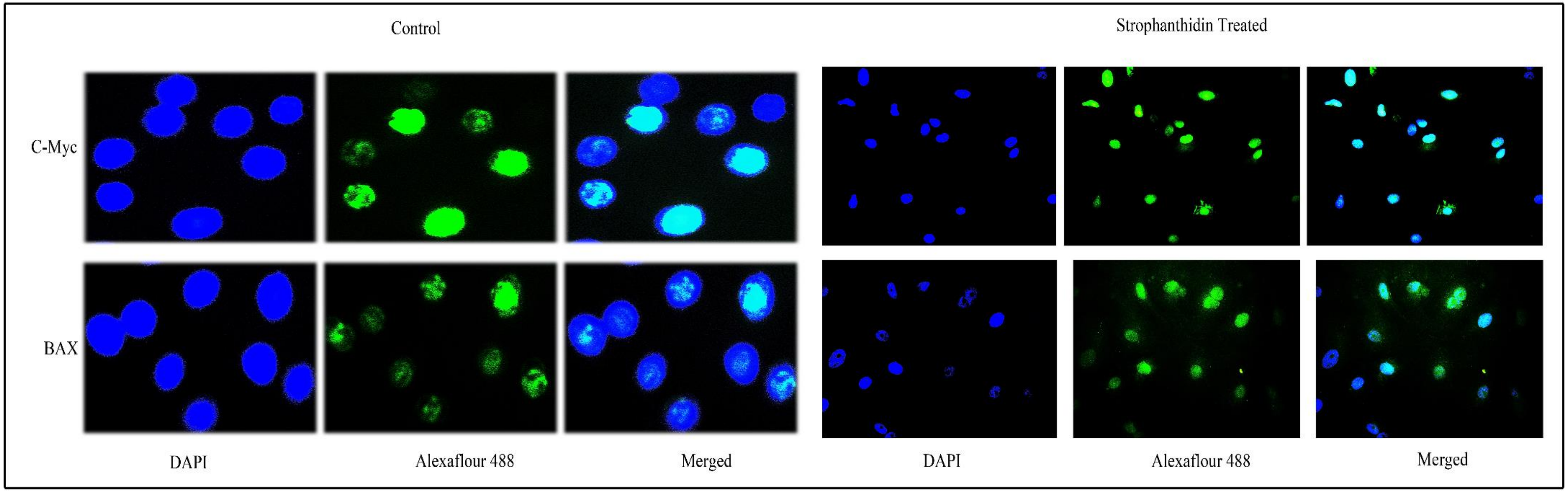

Supplementary Fig. 6D: Immunofluorescence imaging for the analysis of protein localisations in c-Myc and BAX in Strophanthidin induced HepG2 cells.

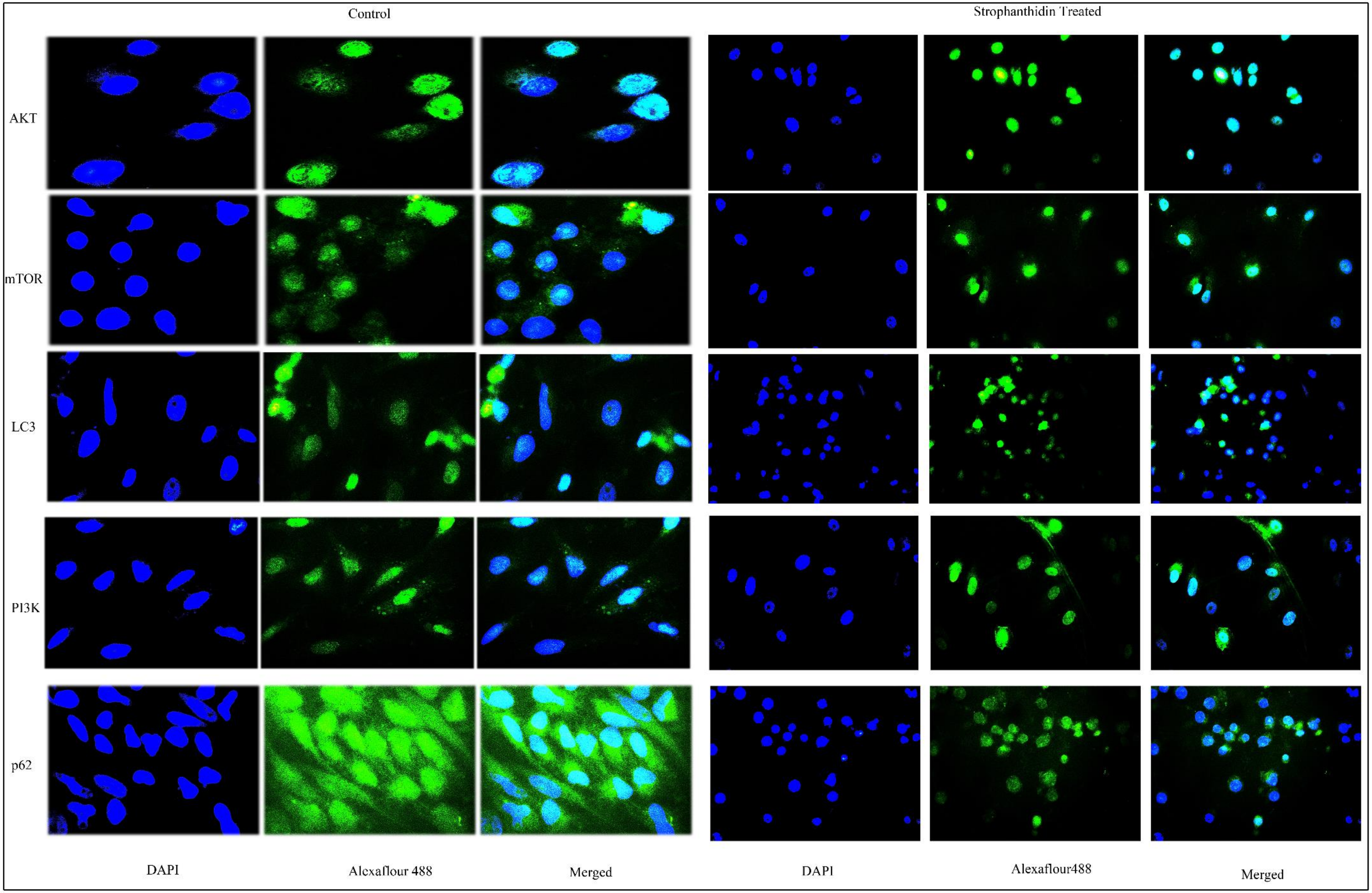

Supplementary Fig. 6E: Immunofluorescence imaging for the analysis of protein localisations in AKT, mTOR, LC3, PI3K and p62 in Strophanthidin induced HepG2 cells.

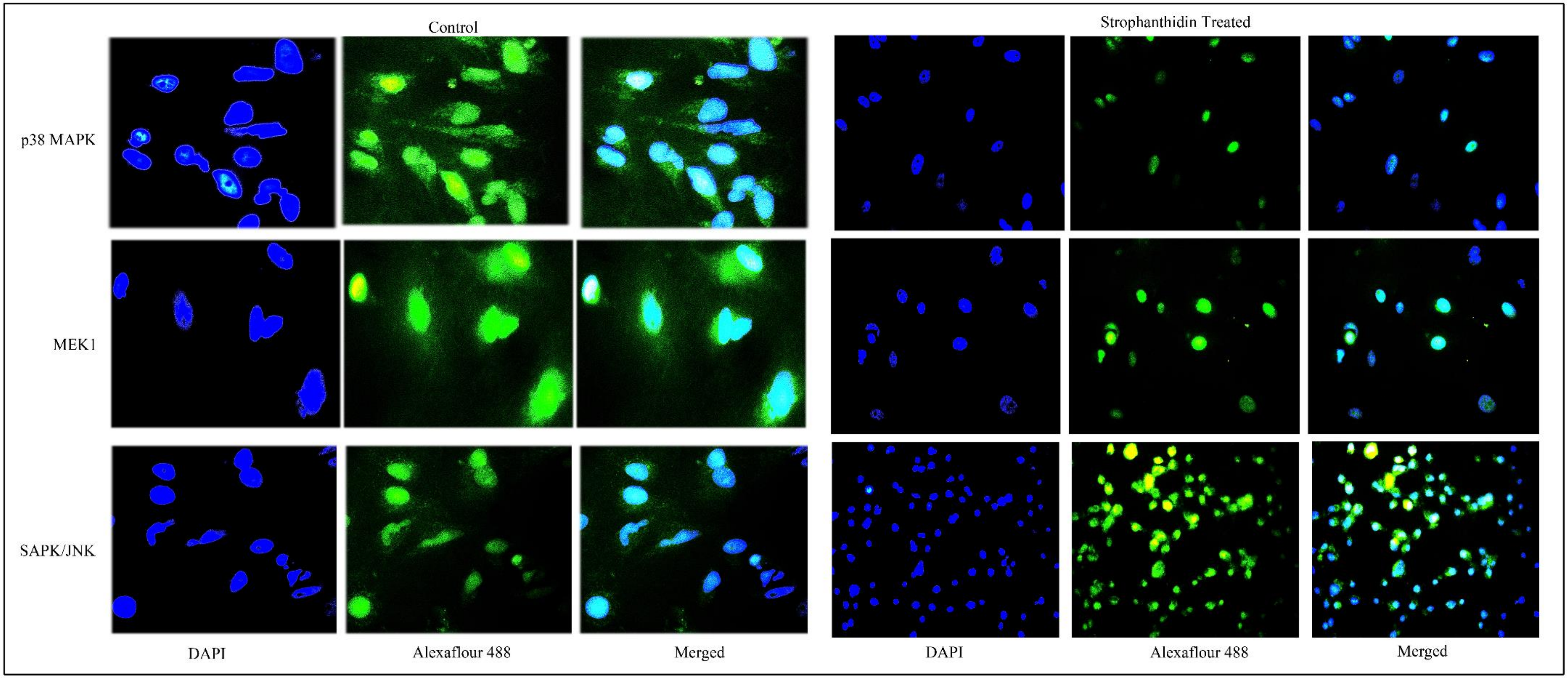

Supplementary Fig. 6F: Immunofluorescence imaging for the analysis of protein localisations of p38MAPK, MEK1and SAPK/JNK in Strophanthidin induced HepG2 cells.

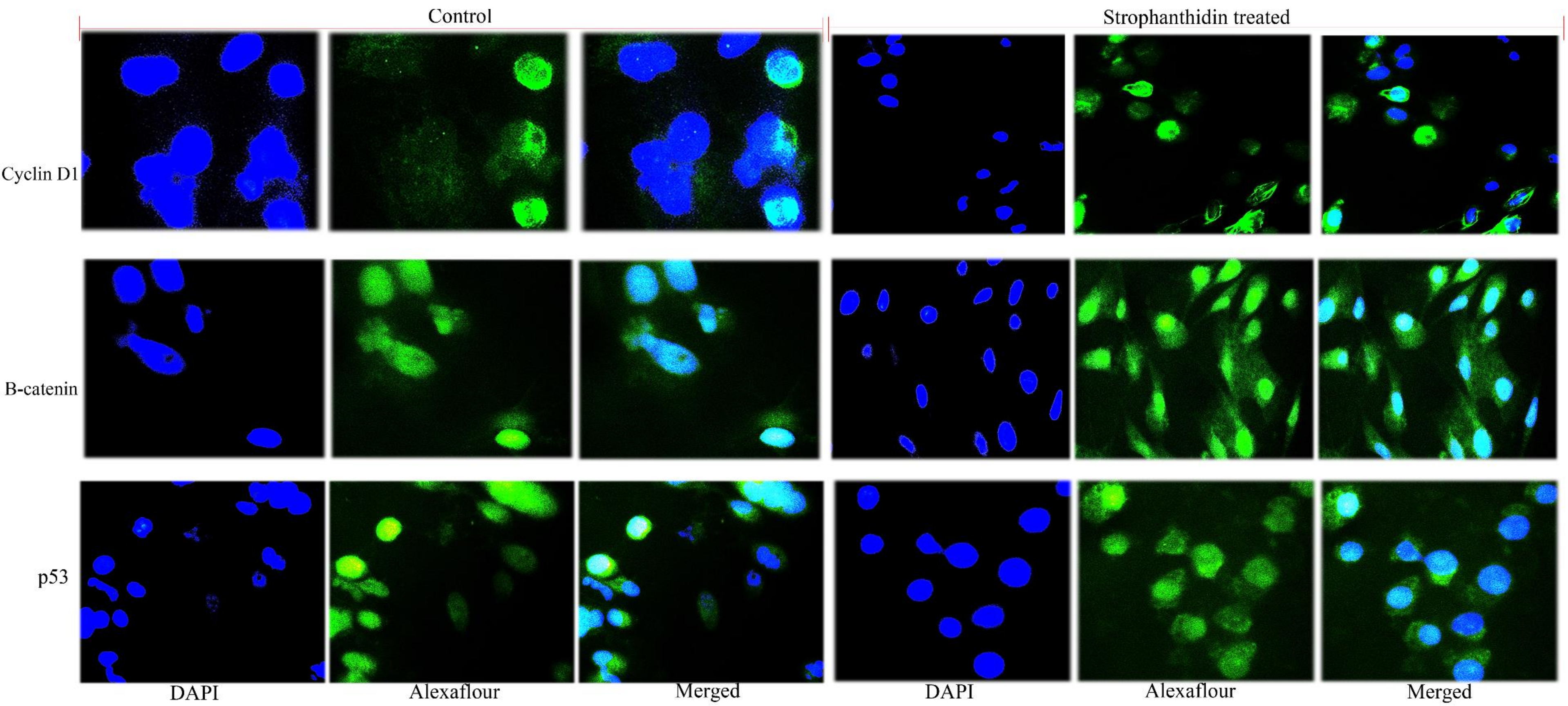

Supplementary Fig. 6G: Immunofluorescence imaging for the analysis of protein localisations of Cyclin D1, β-catenin and p53 in Strophanthidin induced HepG2 cells.

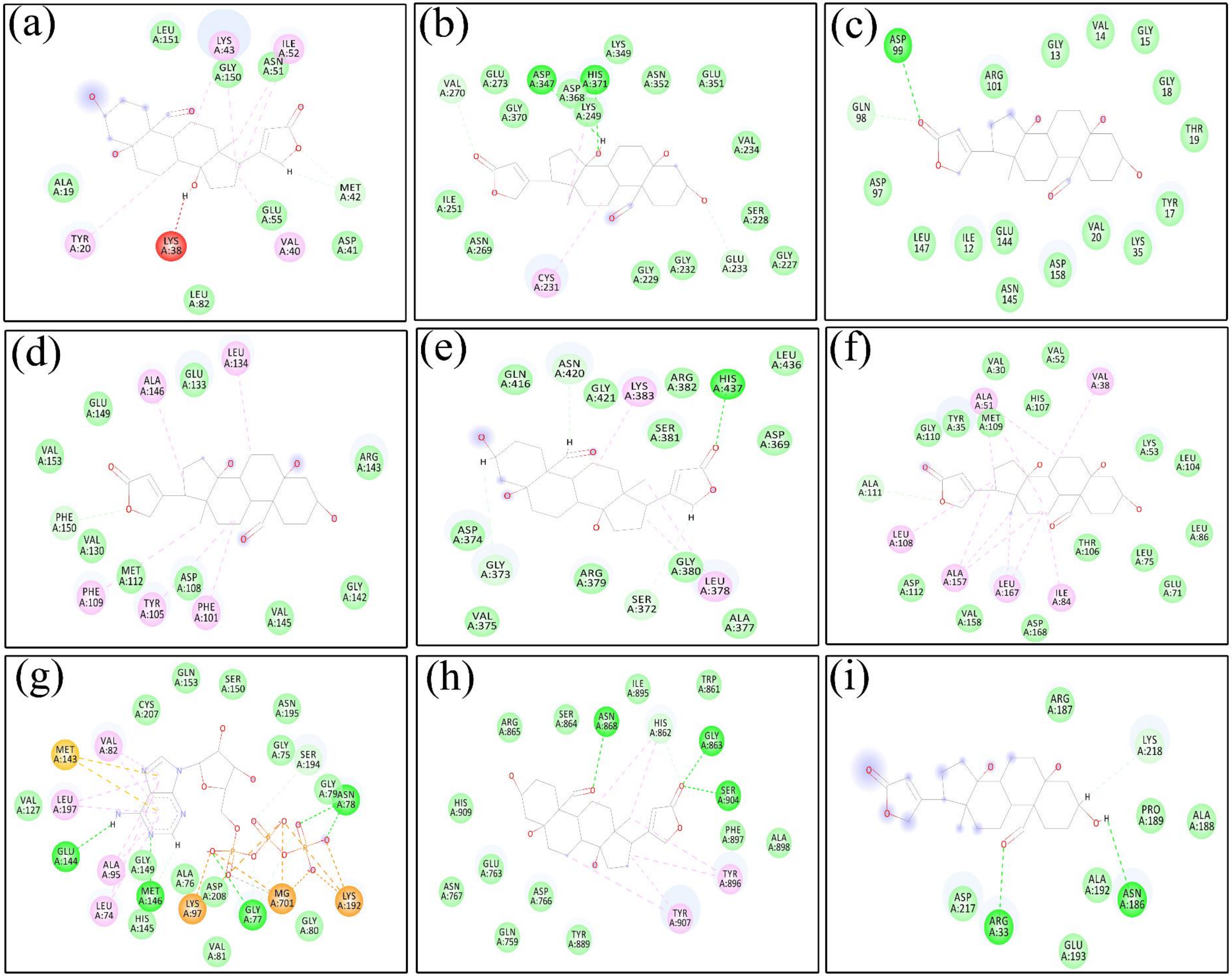

Supplementary Fig. 7: 2D interactions of docked protein and ligand (a) Chk1, (b) Chk2, (c) Cyclin D1, (d) Bcl2, (e) Stat3, (f) p38MAPK, (g) MEK1, (h) PARP and (i) NF-kβ
